# Elucidation of Functional Markers from *Aspergillus nidulans* Developmental Regulator FlbB and Their Phylogenetic Distribution

**DOI:** 10.1371/journal.pone.0017505

**Published:** 2011-03-10

**Authors:** Marc S. Cortese, Oier Etxebeste, Aitor Garzia, Eduardo A. Espeso, Unai Ugalde

**Affiliations:** 1 Department of Applied Chemistry, Faculty of Chemistry, University of the Basque Country, San Sebastián, Spain; 2 IKERBASQUE, Basque Foundation for Science, Bilbao, Spain; 3 Department of Cellular and Molecular Medicine, Centro de Investigaciones Biológicas (CSIC), Madrid, Spain; Institute of Developmental Biology and Cancer Research, France

## Abstract

*Aspergillus nidulans* is a filamentous fungus widely used as a model for biotechnological and clinical research. It is also used as a platform for the study of basic eukaryotic developmental processes. Previous studies identified and partially characterized a set of proteins controlling cellular transformations in this ascomycete. Among these proteins, the bZip type transcription factor FlbB is a key regulator of reproduction, stress responses and cell-death. Our aim here was the prediction, through various bioinformatic methods, of key functional residues and motifs within FlbB in order to inform the design of future laboratory experiments and further the understanding of the molecular mechanisms that control fungal development. A dataset of FlbB orthologs and those of its key interaction partner FlbE was assembled from 40 members of the Pezizomycotina. Unique features were identified in each of the three structural domains of FlbB. The N-terminal region encoded a bZip transcription factor domain with a novel histidine-containing DNA binding motif while the dimerization determinants exhibited two distinct profiles that segregated by class. The C-terminal region of FlbB showed high similarity with the AP-1 family of stress response regulators but with variable patterns of conserved cysteines that segregated by class and order. Motif conservation analysis revealed that nine FlbB orthologs belonging to the Eurotiales order contained a motif in the central region that could mediate interaction with FlbE. The key residues and motifs identified here provide a basis for the design of follow-up experimental investigations. Additionally, the presence or absence of these residues and motifs among the FlbB orthologs could help explain the differences in the developmental programs among fungal species as well as define putative complementation groups that could serve to extend known functional characterizations to other species.

## Introduction

*Aspergillus nidulans* is a filamentous ascomycete that belongs to the subphylum of Pezizomycotina (previously known as Euascomycotina). Although only pathogenic for immunosupressed individuals, it is closely related to other Pezizomycetes of importance in the areas of medicine (e.g., *A. fumigatus*), industry (e.g., *A. niger* and *A. oryzae*) and agriculture (e.g., *A. flavus*). Because of these close relationships and its ease of manipulation in the laboratory, it has been used worldwide as a model organism for more than sixty years [1]. Many basic eukaryotic developmental mechanisms have been revealed in this model organism through the application of genetic, molecular, physiological and biochemical approaches [2].

The life cycle of *A. nidulans*, consisting of three main phases: vegetative extension and asexual and sexual reproduction, has been extensively described in the literature (see references within [3], [4]). These programs require the generation, according to sophisticated developmental pathways, of a set of specialized cell types. Vegetative cells or hyphae are tubular syncytia that grow exclusively by polarized extension [5] through the deposition of new material at the tip [6]. Changes in environmental conditions, mainly the exposure to the atmosphere and light, but also nutritional and abiotic stresses, induce the generation of asexual spores called conidia [7]–[9]. These propagules are generated and dispersed in large quantities from asexual microstructures called conidiophores. The architecture of the conidiophore involves the synthesis of five specialized cell types [10]. First, the foot-cell is generated in distal cells of specific vegetative hyphae. The foot-cell acts as the base of the second cell type, the stalk, which arises and forms an apical swelling or vesicle. Thirdly, a layer of approximately 60 metulae emerges and, after their division, two phialides per metulae. This fourth cell type is the conidia producing structure [11]. Each phialide can produce a chain of more than 100 conidia. Thus, the architecture of each conidiophore allows for the production of more than 10,000 asexual spores, resulting in an efficient dispersive mechanism.

The transduction of environmental cues into intracellular signals that activate the above described morphological transformations is controlled by a signaling cascade in which the transcription factor (TF) FlbB and its interaction partner FlbE play key roles. Deletion of either protein results in a distinctive phenotype characterized by the formation of cottony colonies (‘fluffy’ phenotype) with a broad delay in the timing of conidiation and a substantial reduction in the number of conidiophores with respect to the wild type strain [12], [13]. FlbB interacts with FlbE at the region that sustains vegetative growth, the hyphal tip [13]. FlbB is the only known TF showing such a localization in *Aspergillus nidulans* [14], [15]. The association of FlbB and FlbE is thought to form part of an environmental sensing mechanism that transduces signals to nucleus [14], where FlbB purportedly activates, in conjunction with additional regulators, the genetic pathway that controls the morphological changes required for conidiophore development [16].

However, further progress towards understanding the roles of FlbB requires uncovering the functional determinants encoded within its sequence. In this paper, we apply *in silico* approaches with the goal of associating particular aspects of FlbB functionality with specific motifs and residues in order to inform the design of site-directed *flbB* mutational strategies. Furthermore, analysis across multiple genomes facilitates the identification of functional determinants gained, lost or modified as species evolved independently. This opens the discussion on how far the functional phenotype of the partially characterized *A. nidulans* FlbB protein penetrates into the Pezizomycotina and which specific functions could be shared with orthologs in other species. Finally, we apply a method to discern co-conservation of motifs that could lead to the identification of the site of interaction between FlbB and FlbE.

## Results

### Initial characterization of *A. nidulans* FlbB and FlbE

**Figure 1:**
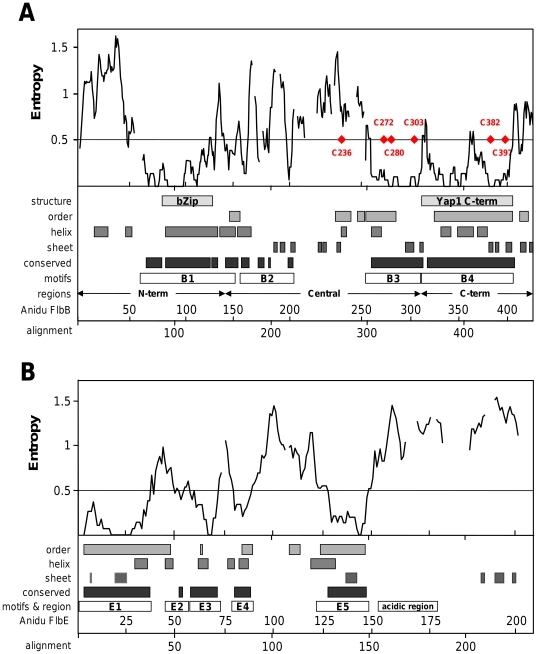
Sequence conservation, structure prediction and motif locations in nine Eurotiales FlbB and FlbE orthologs. The five-column moving average of entropy values for the nine FlbB (A) and nine FlbE (B) alignments (solid line) was plotted. Gaps in the alignment are indicated by the breaks in the moving average line. Regions and motifs are indicated between the plot and x-axes: structural homologies (structure), predicted order (order), predicted secondary structure (helix and sheet) and conserved areas derived from entropy data (conserved) are indicated in increasing shades of grey. The regions selected for further investigation as putative functional motifs (motifs) are indicated by labeled open boxes (B1 – B4, E1 – E5 and ‘acidic region’). The three main domains of FlbB described in the text are indicated above the x-axis. Cysteine locations in FlbB are indicated with red diamonds and labeled with Anidu FlbB residue locations. The x-axes displays both Anidu numbering (above) and alignment numbering (below) to account for the fact that the graphed data is from gapped alignments while the regions and motifs pertain only to Anidu. The total length of the FlbE alignment was 292 residues but only the first 235 residues are included in the graph as only the Pchry extended beyond that.

*Aspergillus nidulans* FlbB is a 426 residue protein that has been the subject of several experimental studies (see references within [16]). The presence of an N-terminal basic region leucine zipper transcription factor domain (bZip) TF domain signature was detected by the National Center for Biotechnology Information (NCBI) Conserved Domain Database search (BRLZ, smart00338) from residues 75 to 126 (Figure 1 A, bZip). Homology to the carboxy-terminal cysteine-rich domain of the TF Yap1, which regulates the response to mild oxidative stress, [17] was established by the Fugue sequence-structure homology recognition server [18] in the C-terminal region of FlbB within residues 311 – 403 (Figure 1 A, Yap1 C-term) with high confidence (Z-score = 10.8). These structural similarities were previously reported [15] although the C-terminal Yap1 similarity was based only on the presence and spacing of cysteines. Between the bZip and C-terminal structured region there is a 165 amino acid central domain with no significant similarities to described motifs or structures.

Extensive database searches with the *A. nidulans* FlbE sequence revealed neither conserved motifs, functional regions nor structural homologies. Previously, it was noted that it contained two conserved regions, a linker between them and an acidic segment in the C-terminal region [13].

We made predictions of order/disorder and secondary structure to further characterize the structural context of conserved areas. Lacking foreknowledge of the complete structural profile of a given protein, order/disorder predictions can help guide choices for mutational strategies and help to uncover regions of functional importance. Both predictions of secondary structure and order/disorder can help in this regard and agreement between the two serves to increases the likelihood that a particular region is structured. For example, conservation in regions of structure (order) could be related to either structure or other functional aspects of the protein. On the other hand, conservation in unstructured (disordered) regions is not likely to be necessary for maintaining structure but can still encode other functions such as protein-protein interactions and regulation (see below for references).

FlbB and FlbE are predicted to be 36% and 43% ordered, respectively, with these regions distributed throughout each protein (Figure 1 A and B, order). The predominant secondary structural type predicted in FlbB was mostly helical with an extended region coinciding with the bZip domain (Figure 1 A, helix). A second region of predicted order that includes both predicted helix and sheet is found in the C-terminal Yap1-like domain (Figure 1 A, order, helix, sheet). Secondary structure prediction for FlbE largely agrees with the order prediction with both sheet and helical regions found in the areas predicted to be structured (Figure 1 B, helix, sheet and order).

The high disordered content of the two proteins (the regions not annotated as ordered) is consistent with their purported signaling and regulatory roles as protein disorder tends to be more prevalent in these types of proteins [19]. There are several attributes of disorder that contribute to its prevalence in protein-protein interactions. For example, energy of binding is reduced for disorder-mediated interactions compared to those mediated by order [20], [21]. This facilitates the reversibility of interactions necessary for dynamic signaling. Other contributions of the disordered state to signaling functionality have been identified experimentally and theoretically [22]–[24].

Preliminary studies of FlbB orthologs in the NCBI database revealed three short conserved sequence motifs that were present only in genomes containing FlbE proteins that were highly similar to *A. nidulans* FlbE (data not shown). In this comparison, the criteria for a credible FlbE was one with an E value less than 7^-43^ and an overlap of greater than 80% with a PSI BLAST [25] profile generated from a Anidu FlbE seed sequence. This analysis compared 19 FlbBs from species with genomes that contained both proteins with 17 genomes that coded for FlbBs but no credible FlbE. In effect, the comparison was between representative sets of Euromycete and non-Euromycete Pezizomycotina. Because the presence or absence of these motifs was linked to the extent that the FlbE ortholog in that genome diverged from the *A. nidulans* FlbE sequence, we wanted to compare the pattern of sequence motif conservation of both proteins across a broad range of fungal species. Potentially, such a study could lead to the elucidation of a set of similarly conserved motifs. Such co-conservation could be due to their mutual interaction. The rationale for this approach is that interacting residues would necessarily be under evolutionary constraints due to the need to maintain compatibility with the corresponding partner motif and would therefore exhibit increased conservation [26]. For this comparison, assembly of data set of genome-paired of FlbB and FlbE proteins was undertaken.

### Construction of FlbB and FlbE datasets

To begin our phylogenetic investigation, we obtained all publicly available sequences with significant similarity to FlbB and FlbE of *A. nidulans* from Pezizomycotina for which both protein sequences were available. Table 1 lists the details of the 40 genome-paired FlbB/FlbE sequences used in this study. Note that the five letter genus/species abbreviations given in the last column of Table 1 will be used from here onward when discussing individual sequences and species (e.g., Anidu for *A. nidulans*).

**Table 1:** Sequences used in this study.

Species and strain	Genomic locus/gi	Protein gi/ID	Protein accession	Source	Abbr.
**FlbB orthologs**					
Ajellomyces dermatitidis ER-3		BDCG_08613		FGI	Aderm
Arthroderma benhamiae CBS 112371		ARB_03643		FGI	Abenh
Aspergillus clavatus NRRL 1		121716128	XP_001275673	NCBI	Aclav
Aspergillus flavus NRRL3357	AFL2G_06507^2^			NCBI	Aflav
Aspergillus fumigatus Af293		302747314	ADL63138	NCBI	Afumi
Aspergillus nidulans		165931814	CAM35586	NCBI	Anidu
Aspergillus niger		145250791	XP_001396909	NCBI	Anige
Aspergillus oryzae RIB40		169775489	XP_001822212	NCBI	Aoryz
Aspergillus terreus NIH2624		115401688	XP_001216432	NCBI	Aterr
Coccidioides immitis RS		CIMG_02371		FGI	Cimmi
Coccidioides posadasii Silveira		CPSG_00217		FGI	Cposa
Cochliobolus heterostrophus C5		81856		JGI	Chete
Fusarium graminearum PH1		FGSG_01313		FGI	Fgram
Fusarium oxysporum		FOXG_00073		FGI	Foxys
Fusarium verticillioides		FVEG_01443		FGI	Fvert
Magnaporthe oryzae 70-15		MGG_00342		FGI	Moryz
Microsporum canis CBS 113480		238844993	EEQ34655	NCBI	Mcani
Microsporum gypseum CBS 118893		MGYG_05249		FGI	Mgyps
Mycosphaerella fijiensis CIRAD86		86007		JGI	Mfiji
Mycosphaerella graminicola		66896		JGI	Mgram
Neosartorya fischeri NRRL 181		119481803	XP_001260930	NCBI	Nfisc
Neurospora crassa OR74A		NCU07379		FGI	Ncras
Neurospora discreta FGSC 8579		166536		JGI	Ndisc
Neurospora tetrasperma FGSC 2508		148703		JGI	Ntetr
Paracoccidioides brasiliensis Pb01		PAAG_01697		FGI	Pb-01
Paracoccidioides brasiliensis Pb03		PABG_03745		FGI	Pb-03
Penicillium chrysogenum Wisconsin 54-1255		255931005	XP_002557059	NCBI	Pchry
Penicillium marneffei ATCC 18224		212538897	XP_002149604	NCBI	Pmarn
Pyrenophora tritici-repentis Pt-1C-BFP		PTRG_07994		FGI	Ptrit
Sclerotinia sclerotiorum 1980		SS1G_08098		FGI	Sscle
Stagonospora nodorum SN15		SNOG_04391		FGI	Snodo
Talaromyces stipitatus ATCC 10500		242820034	XP_002487435	NCBI	Tstip
Thielavia terrestris strain NRRL 8126		36483		JGI	Tterr
Trichoderma atroviride	Tatro_contig_27^2^			JGI	Tatro
Trichoderma reesei		57840		JGI	Trees
Trichoderma virens		15555		JGI	Tvire
Trichophyton rubram CBS 118892		TERG_02140		FGI	Trubr
Trichophyton tonsurans CBS 112818		TESG_03658		FGI	Ttons
Trichophyton verrucosum HKI 0517	NW_003315534^2^			NCBI	Tverr
Verticillium albo-atrum VaMs.102		VDBG_06054		FGI	Valbo
Verticillium dahliae VdLs.17		VDAG_03214		FGI	Vdahl
**FlbE orthologs**					
Ajellomyces dermatitidis ER-3		BDCG_00996		FGI	Aderm
Arthroderma benhamiae CBS 112371	ARB 00079^1^			FGI	Abenh
Aspergillus clavatus NRRL 1	ACLA 021460^1^			FGI	Aclav
Aspergillus flavus NRRL3357	AFL2G 03377^1^			FGI	Aflav
Aspergillus fumigatus		44889993	CAF32111	NCBI	Afumi
Aspergillus nidulans		227433961	ACP28868	NCBI	Anidu
Aspergillus niger	NT_166524^1^			NCBI	Anige
Aspergillus oryzae		281311977	BAI58988	NCBI	Aoryz
Aspergillus terreus NIH2624	NT_165972^1^			NCBI	Aterr
Coccidioides immitis RS		CIMG_03365		FGI	Cimmi
Coccidioides posadasii Silveira		CPSG_02591		FGI	Cposa
Cochliobolus heterostrophus C5	CocheC5_1 scaffold_32^1^			JGI	Chete
Fusarium graminearum PH1		FGSG_09567		FGI	Fgram
Fusarium oxysporum		FOXG_06268		FGI	Foxys
Fusarium verticillioides	FVEG_04121^1^			FGI	Fvert
Magnaporthe oryzae 70-15		MGG_01731		FGI	Moryz
Microsporum canis CBS 113480		238837821	EEQ27483	NCBI	Mcani
Microsporum gypseum CBS 118893		MGYG_01593		FGI	Mgyps
Mycosphaerella fijiensis CIRAD86	Mycfi2 scaffold 6^1^			JGI	Mfiji
Mycosphaerella graminicola	Mycgr3 chr 6^1^			JGI	Mgram
Neosartorya fischeri NRRL 181		119495126	XP_001264355	NCBI	Nfisc
Neurospora crassa OR74A		NCU05255		FGI	Ncras
Neurospora discreta FGSC 8579		141834		JGI	Ndisc
Neurospora tetrasperma FGSC 2508		106945		JGI	Ntetr
Paracoccidioides brasiliensis Pb01		PAAG_02176		FGI	Pb-01
Paracoccidioides brasiliensis Pb03		PABG_02411		FGI	Pb-03
Penicillium chrysogenum Wisconsin 54-1255		255941552	XP_002561545	NCBI	Pchry
Penicillium marneffei ATCC 18224	212534004^1^			NCBI	Pmarn
Pyrenophora tritici-repentis Pt-1C-BFP		PTRG_02296		FGI	Ptrit
Sclerotinia sclerotiorum 1980		SS1G_09830		FGI	Sscle
Stagonospora nodorum SN15		SNOG_03047		FGI	Snodo
Talaromyces stipitatus ATCC 10500	242779542^1^			NCBI	Tstip
Thielavia terrestris strain NRRL 8126		118518		JGI	Tterr
Trichoderma atroviride	Triat2 contig 26^1^			JGI	Tatro
Trichoderma reesei	Trire2 scaffold 11^1^			JGI	Trees
Trichoderma virens	TriviGv29 8 2 scaff 80^1^			JGI	Tvire
Trichophyton rubram CBS 118892	TERG_07460^2^			FGI	Trubr
Trichophyton tonsurans CBS 112818		TESG_06544		FGI	Ttons
Trichophyton verrucosum HKI 0517	TRV_00925^2^			FGI	Tverr
Verticillium albo-atrum VaMs.102		VDBG_00858		FGI	Valbo
Verticillium dahliae VdLs.17		VDAG_00467		FGI	Vdahl

Where genomic locus/gi is given, the protein sequence used was derived from genomic DNA using the following procedures: ^1^The longest continuous ORF encoded by the genomic locus, ^2^Fgenesh prediction.

### Review of gene finding results

Following sequence selection and preliminary analyses, the protein sequences for both sets of orthologs were compared to their genomic loci to check ORF and intron calling. This was necessary because the methodologies that genome projects employ to predict protein sequences vary among organizations and individual projects.

Few changes to FlbB orthologs were necessary. The cDNAs for FlbB of Anidu and Afumi were previously sequenced (NCBI accession numbers CAM35586 and ADL63138, respectively) and these formed a basis for verifying the gene finding results of the remaining sequences. Five intron locations were identified in the FlbB orthologs (Figure S1). The protein sequence for Aflav FlbB in the Broad Institute and NCBI data bases was significantly shorter than other orthologs. Additional genomic sequence encoding an additional 93 amino acids was obtained and translated. The resulting protein was a good match in length and sequence with the closely related Aoryz ortholog. The protein sequence was derived from Aflav AFL2G_06507 (Contig7, 124078-125873) using Fgenesh.

For FlbE, gene finding was complicated by the existence of two alternative transcripts that have been described for Anidu. NCBI protein data base entries ACP28868 and CAP08290 document intronless and intron forms, respectively, that are both conceptual translations from sequenced cDNA. Examples of alternative splicing, although rare, have been documented in Aspergilli [27]. The relationship between the alternative forms and FlbE function has not been explored experimentally. The alternative transcripts have different sequence from residue 186 to the C-terminus. This region of Anidu FlbE is outside all conserved regions and those shown to have functional roles [13], [28]. The possibility that FlbE could be subject to alternative splicing [29] opens the question as to which form should be used in our study. The fact that the only other experimentally derived FlbE sequence, Aoryz NCBI accession BAI58988, is intronless, supports using the intronless transcript forms, at least for those species closely related to Anidu and Aoryz. Thus, all Eurotiales FlbE sequences were derived from the longest continuous ORF encoded by the respective genomic sequence.

In general, this intronless nature held for the rest of the orthologs in spite of introns being predicted in 20 of the original FlbE sequences obtained from genome project and NCBI databases. The purported intron-containing FlbEs exhibited a scattered phylogenetic distribution with abundant inconsistencies in splice site locations. However, support was found for one cluster of FlbEs with a single intron in the Oxygenales (Ttons, Trubr, Tverr, Mgyps and Mcani; Figure S2). For the remaining sequences, support was for an intronless nature. Evidence for support of either outcome included: conserved intron locations in closely related sequences and phylogenetic consistency in terms of homology. Thus, the net effect of the changes made during this process was improvement of the quality of the alignment.

### Evaluation of structural homologies in full data set

To evaluate the structural similarities that were found in the experimentally characterized Anidu FlbB sequence in the remaining orthologs, each was evaluated for presence of a N-terminal bZip domain by searching the SMART database [30] and for C-terminal homology to the carboxy-terminal cysteine-rich domain of the Yap1 TF [17] using the Fugue sequence-structure homology recognition server [18]. The bZip signature was identified at significant E values in every FlbB sequence except Pmarn and Tstip where values were less than significant since both diverge noticeably from the consensus motif in the later part of the domain. As for the homology to the C-terminal region of Yap1, all FlbB orthologs were deemed to have homology equal to or greater than 90% confidence by Fugue except Ntetr and Ncras, which were scored at 50% confidence. These results, along with the facts that they were approximately the same length (396±31 amino acids) and exhibited substantial amounts of identity and similarity throughout the length of the aligned sequences (Figure S1), suggested that all 40 were orthologous. High levels of identity and similarity were also observed among the FlbE sequences (Figure S2).

**Figure 2:**
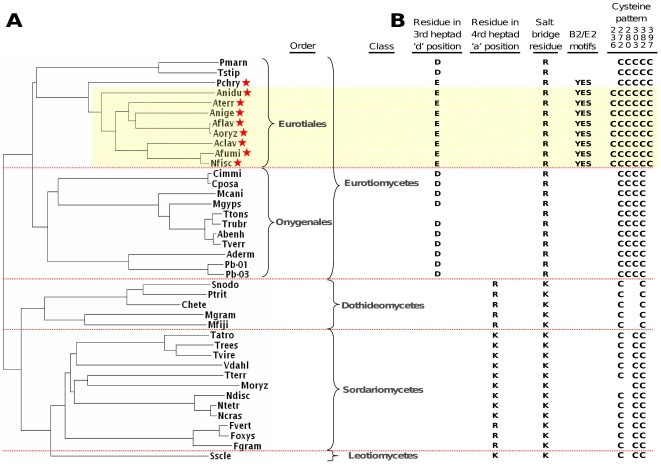
Phylogenetic tree and differential features of 40 Pezizomycotina FlbB orthologs. A. Phylogenetic tree of FlbB orthologs generated from pairwise CLUSTAL distances. Four clades equivalent to the fungal classes Eurotiomycetes, Dothideomycetes, Sordariomycetes and Leomycetes are labeled. Two order level subclades within the Eurotiomycetes are also labeled: the Eurotiales and Oxygenales. The nine Eurotiales used for conserved motif discovery are starred. The eight species with putative functionally equivalent FlbB proteins are shaded in yellow. B. Differential functionally-related features identified in the sequences as described in the text. The presence of the six conserved cysteines are denoted by ‘C’ in the column labeled according to Anidu numbering. ‘Yes’ indicates that both the B2 and E2 motifs are present in that species.

The topology of the phylogenetic tree generated from the FlbB CLUSTAL alignment did not differ substantially from previously published fungal phylogenies (Figure 2 A)[31]–[33], suggesting that FlbB evolved unremarkably. The topology of the FlbE tree was quite similar to the FlbB tree with no unusual deviations (data not shown).

### Conservation of critical bZip residues

Having established that bZip domains were present in 40 FlbB orthologs, we next determined the level of conservation of bZip signature residues. The generalized consensus sequence for the bZip DNA binding domain (DBD) is **N**[RK]x[**AS**][**ASQ**]xx**[SCFY**]**R**, with the two underlined residues being invariant and the bold residues contacting the DNA [34], [35]. The two invariant residues were present in all 40 DBD-containing FlbB orthologs. Furthermore, taking all the conserved residues into account (Figure S1, purple highlight), the bZip DBD subfamily most closely related to FlbB orthologs is the PAP subfamily (consensus sequence NxxAQxx**F** R) [35]. The FlbB orthologs, however, match all but one of the five DNA-contacting residues in the PAP signature with the phenylalanine (F, in bold) being substituted with histidine (H) in all 40 orthologs giving the consensus sequence NxxAQxx**H** R for the FlbB DBD (Figure S1). The fact that all 40 DBD-containing FlbB orthologs contain this histidine suggests that it is important and specific for FlbB function. Although much effort has been expended in the study of bZip transcription factors including comprehensive reviews of the multiple subfamilies [35]–[38], this histidine-containing DBD consensus has not been described. We designated this particular DBD ‘H19’ in keeping with a systematic designation for describing substitutions in this position [37] (see discussion).

As this H19 bZip DBD appeared to be novel, we conducted an exhaustive search of public and sequencing project databases to determine the extent of its distribution. The prevalence of the H19 bZip DBD was limited with one in *Pichia stipitis* (subphylum Saccharomycotina), five within fungi but outside Ascomycota, one in phylum Oomycota and one in kingdom Viridiplantae (Table 2). This limited distribution outside Pezizomycotina further supports the hypothesis that the histidine of H19 is important and specific for FlbB function.

**Table 2:** H19 bZip DNA binding domains found outside Pezizomycotina.

Organism	Kingdom	Phylum	Subphylum	Order	Source	Accession or reference
Pichia stipitis	Fungi	Ascomycota	Saccharomycotina	Saccharomycetales	NCBI	gi|150951570|ref|XP_001387909.2|
Coprinopsis cinerea	Fungi	Basidiomycota		Agaricales	NCBI	gi|299746530|ref|XP_001838046
Cryptococcus neoformans	Fungi	Basidiomycota		Tremellales	NCBI	gi|134110800|ref|XP_775864.1|
Laccaria bicolor	Fungi	Basidiomycota		Agaricales	NCBI	gi|170105495|ref|XP_001883960
Postia placenta	Fungi	Basidiomycota		Polyporales	JGI	jgi|Pospl1|129836|estExt_fgenesh3_pg.C_1080015
Schizophyllum commune	Fungi	Basidiomycota		Agaricales	NCBI	gi|300105220|gb|EFI96625.1|
Phytophthora infestans	Stramenopila	Oomycota		Peronosporales	NCBI	gi|262097289|gb|EEY55341.1|
Volvox carteri	Viridiplantae	Chlorophyta		Chlamydomonadales	NCBI	gi|300255540|gb|EFJ39839.1|

**Figure 3:**
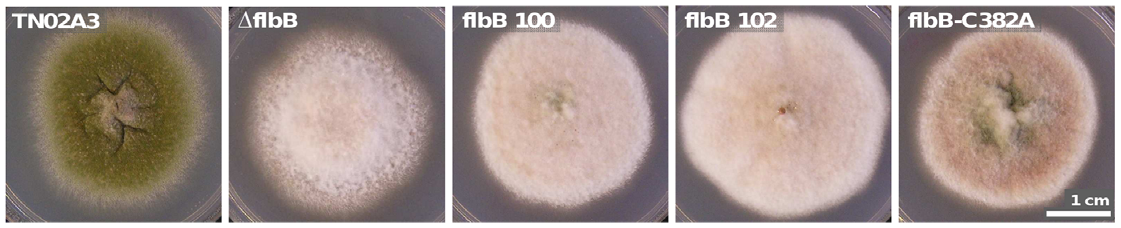
Characterization of FlbB-C382A conidiation phenotype. Condidial phenotype of the parental wild type TN02A3 compared to mutant strains ΔflbB, flbB 100, flbB 102 and flbB-C382A. FlbB produced by flbB 100 is truncated after amino acid P305 and that of flbB 102 has a G70R substitution.

We have previously demonstrated that the region immediately N-terminal to the highly conserved bZip core is also important for bZip function. The flbB100 allele was identified in a random mutagenesis that sought aconidial (“fluffy”) mutations [15]. This flbB mutant allele encodes for a FlbB protein with a change from glycine to arginine at position 70 (G70R). Coincident with the inability to produce conidiospores on Aspergillus Minimal Medium (Figure 3), the G70R mutation results in a remarkable decrease in the capability of the FlbB bZip to bind previously defined DNA targets compared to the wild type protein [14].

Both homo- and hetero-dimerizaton of bZip TFs are possible with the dimerization interface composed of leucine repeats located immediately C-terminal to the DBD [37], [39]. This interface typically contains four to five heptad repeats that mediate binding between two compatible monomers via formation of a coiled-coil. By convention, the residues within each heptad are labeled ‘a’ through ‘g’ [40]. A leucine zipper is formed primarily by hydrophobic residues in the ‘a’ and ‘d’ positions of each heptad. These residues form a hydrophobic interface in which each position of one monomer interacts with its counterpart in the other monomer. However, non-hydrophobic residues, which serve to modify the specificity of the interface, can also be found in these positions [38].

Contrary to the homogeneity found in the DBD, inspection of the CLUSTAL alignment of the 40 orthologs (Figure S1) revealed that gaps were present in the bZip dimerization domain, indicating that insertions and/or deletions had occurred in this region as the Pezizomycotina evolved. However, the first three heptads and the ‘a’ residue of the fourth heptad are in a gap-less region adjoining the DBD and were sufficient to differentiate two different dimerization profiles among the orthologs.

Lysines or arginine in the ‘a’ or ‘d’ positions function to promote hetero- and disfavor homo-dimerization [38]. As the ‘a’ and ‘d’ residues associate with their counterparts along the hydrophobic interface, charged residues in these positions are thought to be a mechanism to disfavor homodimerization [38], [41], [42]. We know of no studies on the implications of negatively charged residues in the ‘d’ position but, similar to positive charges in these positions, a similar bias against homo-dimerization would be expected. In Anidu FlbB, the ‘d’ position of the third heptad is glutamic acid (Glu). This negative charge modifies the hydrophobic character of the dimerization interface and likely has a strong influence on dimerization partner selection. Significantly, all of the Eurotiomycetes have a charged residue in this position except Ttons, which has a glycine (Figure 2 B; Figure S1 orange highlight). The most common residue in this group is aspartic acid (Asp) with Glu occurring in eight orthologs. The evolutionary relationship between the Asp- and Glu-substituted orthologs is clearly defined with the Asp- and Glu-containing groups clustering separately (Figure 2). However, as the charge is conserved between the two possible substitutions, there may be no substantial differences between the dimerization profiles of the Asp- and Glu-containing orthologs.

In contrast, the 19 non-Eurotiomycete orthologs lack this negative charge and instead contain a positively charged residue in the ‘a’ position of the fourth heptad (Figure 2 B; Figure S1, orange highlight). Similar to the conservative substitutions observed for the ‘d’ position of the third heptad, this charge could be either an arginine (Arg) or a lysine (Lys). This conservative substitution implies the same low likelihood of substantially altered dimerization properties. However, unlike the differential substitutions of the negatively charged residue in the third heptad, the evolutionary relationships among those containing this positive residue are not straightforward. All five Dothideomycetes contain Arg and the single Leotiomycete contains Lys. On the other hand, the Sordariomycetes are mixed, with most containing Lys but with a middle branching group composed of Fgram, Fvert and Foxys containing Arg (Figure 2, Figure S1). The possibility that the conservative Lys->Arg substitution arose more than once supports the supposition that no radical changes in dimerization properties are associated with these like-charged substitutions.

In some bZips, a second mechanism can influence dimerization properties. Both homo- and hetero-dimers can be stabilized by salt bridge formation between oppositely charged residues in the ‘g’ position of one heptad in one monomer and the ‘e’ position of the following heptad in the other monomer [41]. On the other hand, non-complementary charges in these positions disfavor dimerization [42]. In the dimerization domains of the 40 orthologs, a salt bridge is predicted between the ‘g’ residue of the first heptad and the ‘e’ residue of the second heptad (Figure 2 B; Figure S1, light blue highlight). The negatively charged residue of the salt bridge (in the ´ǵposition of the first heptad) is a completely conserved glutamic acid (Glu). According to their respective positions within the leucine zipper heptads, this residue is predicted to form a salt bridge with arginine (Arg) in all the Eurotiomycetes and lysine (Lys) in the Dothideomycetes, Sordariomycetes and Leotiomycetes orthologs (Figure 2; Figure S1, light blue highlight). As the positive charge is conserved with either amino acid, it is likely that the same salt bridge functionality exists in all the orthologs.

### C-terminal structured region of FlbB

As discussed above, the C-terminal region of the 40 orthologs have homology to the C-terminal redox responsive domain of Yap1. PSI-BLAST, CLUSTAL, HMMalign and Fugue all align Anidu FlbB cysteine C382 with C598 of Yap1 and C501 of Pap1 (data not shown), both of which are implicated in the redox modulation of stability and localization of Yap1 [43] and Pap1 [44]. Both proteins are bZip TFs that translocate between the cytosol and nucleus depending on the redox status of the cell. According to the CLUSTAL alignment, all 40 FlbB orthologs contain this cysteine (hereafter referred to as C382; Figure S1, red highlight) which, moreover, is within a group of six highly conserved residues. In both Yap1 and Pap1, the positionally equivalent cysteine participates in intramolecular disulfide bond formation in response to oxidative stress by forming bonds with either a C-terminal or a central region cysteine depending on the level of oxidative stress [17], [43], [44].

As C382 was conserved in all 40 FlbB orthologs, we sought to verify that this cysteine was critical for FlbB function in Anidu. A mutant strain expressing FlbB with cysteine 382 substituted by an alanine (C382A) was constructed. The phenotype of the FlbB C382A strain was analysed in relation to the parental wild type, an *flbB* null strain and *flbB102* (truncated at amino acid 305) [15] (Figure 3). flbB-C382A exhibited a fluffy phenotype with sparse conidiation thus confirming that C382 is critical for FlbB function in Anidu.

**Figure 4:**
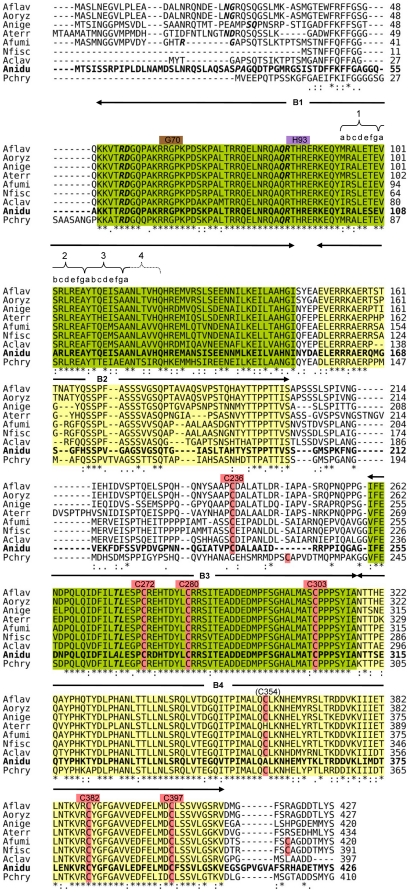
Alignment of the nine Eurotiales FlbB orthologs used to generate HMM motif profiles. CLUSTAL alignment of the nine Eurotiales FlbB orthologs used to generate conserved motifs. Anidu FlbB is in bold. Motifs B1, B2, B3 and B4 are labeled above the alignment and highlighted in green or yellow. The heptads of the bZip dimerization domain (the first three heptads and the first residue of the third) are identified by brackets with the residue positions labeled a – g according to convention. Cysteine residues are labeled with Anidu numbering and highlighted in light red. The positions of G70 and H93 in the bZip DBD (Anidu numbering) are also labeled. Residues flanking intron locations are in bold italic.

In light of this experimental result and the homology to Yap1, C382 in the FlbB orthologs likely comprises one half of a di-sulfide bond forming pair. Hence, the question arises: Which other FlbB cysteines could form bonds with C382? Based entirely on conservation and the assumption that the FlbB orthologs do, in fact, undergo intramolecular disulfide bond formation, the most likely partner for C382 would be C272 (Figure 2 B, Figure 4, Figure S1). (Note that Anidu numbering will be used whenever residue positions are reported.)

The remaining cysteines exhibit varying levels of conservation with distinct patterns that segregate according to class and order. The presence or absence of the six most relevant conserved cysteines (C236, C272, C280 and C303 in the central region and C382 and C397 in the C-terminal region) are indicated for each species in Figure 2 B. An additional potentially relevant cysteine, C354 in Anidu numbering (Figure 4, Figure S1), is conserved in seven of the Eurotiales but was not considered here for two reasons. First, it is not found in any of the orthologs outside the Eurotiales and therefore does not contribute phylogenetic information to the study of Anidu FlbB. Second, it is not present in Anidu and therefore does not have a role in the characterized function of Anidu FlbB and thus has no impact on the transfer of experimentally derived information from that species to the other orthologs. Eight Eurotiales, including Anidu, contain all six of the most conserved cysteines (Figure 2; Figure 4 and Figure S1, red highlight). In the other Eurotiales (Pmarn, Tstip and Pchry), five of these cysteines are completely conserved. In the Onygenales there are four completely conserved cysteines, with C236 and C397 missing in all the sequences. There are only two completely conserved cysteines in the Dothideomycetes, C272 and C382, providing additional support for this pair being redox active. All Sordariomycetes have C303, C272 and C382 except Moryz which is missing C272.

### Extracting conserved motifs from FlbB and FlbE

In order to identify potential functional determinants and clues to potential coevolving interaction sites, we set out to identify the conserved regions of FlbB and FlbE. Although conserved regions were observed in the alignments of both sets of orthologs (Figure S1; Figure S2), we choose to map areas of conservation within a cohort of nine species most closely related to Anidu. This selection would be more likely to include conserved regions that encoded some of the experimentally characterized functionality of Anidu FlbB. These sequences were chosen based on BLAST scoring with each FlbB ortholog having greater than 60% identity and an E value less than 1^-120^ and each FlbE ortholog having greater than 50% identity and an E value less than 1^-50^. Additionally, the selected sequences were genome-paired. Not surprisingly, the nine species were members of the Eurotiales subclade (Figure 2, red stars).

FlbB motifs were selected based on structural information and entropy analysis. To this end, entropy was calculated for each column (residue position) of the alignments of the nine Eurotiales orthologs (Figure 4) and a 5-residue moving average was calculated (Figure 1, Entropy). Initially, conserved motifs were defined as those regions with a moving average of entropy less than 0.5. The motifs were expanded as necessary to include: structural features (bZip and Yap1 C-term similarities), columns with conservative substitutions, columns with high levels of conservation that were not reflected in the moving averages and to combine short stretches of conservation into larger fragments (Figure 1 A, conserved and motifs). For example, B2 is comprised of a cluster of four smaller conserved regions. B3 and B4 are contiguous but we chose to terminate B3 at the beginning of B4, which was defined by structural homology to Yap1 PDB structure 1SSE as determined by Fugue. In all, four motifs (B1–B4) were identified in the FlbB alignment of the nine Eurotiales (Figure 1 A, Figure 4, Table 3).

**Table 3:** Length and location of regions and motifs in Anidu FlbB and FlbE.

Protein	Designation	Length (AA)	Residues (Anidu numbering)
Anidu FlbB			
	N-term	144	1–144
	Central	166	145–310
	C-term	116	311–426
	B1	96	57–152
	B2	47	157–203
	B3	58	253–310
	B4	95	311–405
Anidu FlbE			
	E1	41	1–41
	E2	13	45–57
	E3	16	58–73
	E4	12	79–90
	E5	29	121–149
	Acidic region	25	153–177

**Figure 5:**
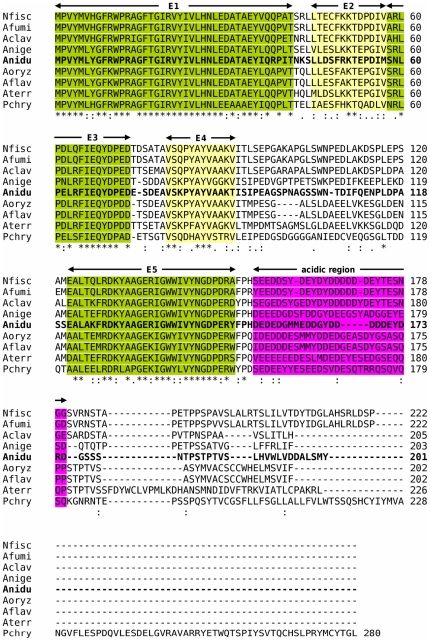
Alignment of the nine Eurotiales FlbE orthologs used to generate HMM motif profiles. CLUSTAL alignment of the nine Eurotiales FlbE orthologs used to generate conserved motifs. Anidu FlbE is in bold. Motifs E1, E2, E3, E4 and E5 are highlighted in green or yellow. The acidic region is highlighted in purple.

As no conserved domains or motifs were found when the Anidu FlbE sequence was used to search conserved domain databases, motifs E1–E5 were based solely on entropy data (Figure 1 B, Figure 5, Table 3). Additionally, FlbE orthologs were evaluated for the existence of an acidic region C-terminal to motif E5 that had been previously noted [13]. Since there was little positional conservation in this region, it was scored by calculating average charge (see Methods section). An acidic region was present in all the Eurotiomycetes except Pb-01, Pb-02 and 11 other species dispersed among the Dothideomcetes, Sordariomycetes and Leotiomycetes (Table 4).

**Table 4:** HMM profile scores and other parameters for FlbB and FlbE orthologs.

		FlbB						FlbE							
			HMM profile E values						HMM profile E values						
Order	Species abbr.	Length	Full length	B1	B2	B3	B4	Length	Full length	E1	E2	E3	E4	E5	Acidic Region*
**Eurotiales**	**Aflav**	**427**	4.2^-265^	3.4^-62^	3.0^-27^	2.7^-46^	9.6^-65^	**202**	1.3^-111^	9.3^-35^	1.0^-8^	1.8^-13^	9.6^-8^	7.3^-25^	**YES**
**Eurotiales**	**Aoryz**	**427**	1.4^-264^	3.5^-62^	2.5^-27^	3.0^-46^	8.9^-65^	**202**	1.3^-111^	9.3^-35^	1.0^-8^	1.8^-13^	9.6^-8^	7.3^-25^	**YES**
**Eurotiales**	**Aterr**	**434**	1.3^-254^	8.7^-62^	5.6^-28^	4.1^-46^	5.2^-62^	**226**	9.1^-115^	2.3^-35^	4.4^-8^	2.4^-13^	7.0^-8^	9.5^-25^	**YES**
**Eurotiales**	**Anige**	**420**	1.2^-252^	6.8^-62^	1.4^-27^	1.2^-45^	8.3^-64^	**203**	7.6^-111^	3.5^-35^	5.4^-8^	4.0^-14^	5.8^-8^	4.0^-25^	**YES**
**Eurotiales**	**Afumi**	**420**	8.4^-251^	1.7^-60^	9.6^-28^	2.9^-46^	9.2^-65^	**222**	2.2^-118^	2.5^-35^	2.2^-9^	1.6^-13^	5.8^-8^	1.2^-24^	**YES**
**Eurotiales**	**Nfisc**	**391**	4.1^-244^	6.9^-61^	7.0^-28^	5.0^-46^	1.1^-64^	**222**	2.4^-118^	5.7^-35^	2.2^-9^	1.6^-13^	5.7^-8^	1.5^-24^	**YES**
**Eurotiales**	**Anidu**	**426**	1.5^-235^	2.6^-59^	3.2^-25^	2.7^-45^	2.1^-61^	**201**	2.2^-104^	6.4^-35^	2.2^-8^	1.6^-13^	9.4^-8^	9.4^-24^	**YES**
**Eurotiales**	**Aclav**	**397**	7.8^-241^	1.5^-60^	3.5^-23^	6.0^-46^	1.6^-64^	**205**	5.4^-115^	1.2^-34^	2.3^-9^	3.2^-14^	4.9^-8^	9.7^-25^	**YES**
**Eurotiales**	**Pchry**	**410**	8.0^-230^	3.8^-57^	9.0^-25^	9.1^-46^	1.3^-60^	**280**	1.7^-104^	4.5^-34^	1.2^-7^	8.2^-13^	9.4^-8^	1.3^-21^	**YES**
Onygenales	Cposa	434	2.3^-143^	3.6^-47^		6.7^-25^	3.0^-44^	187	1.3^-65^	4.2^-17^		1.2^-9^	6.6^-5^	6.6^-17^	YES
Onygenales	Cimmi	434	2.5^-143^	3.6^-47^		6.6^-25^	3.0^-44^	187	1.7^-65^	4.8^-17^		1.2^-9^	6.5^-5^	6.6^-17^	YES
Eurotiales	Pmarn	431	1.0^-136^	1.6^-43^		7.4^-26^	8.4^-48^	208	3.8^-63^	4.5^-20^		6.0^-8^	2.2^-4^	1.4^-17^	YES
Eurotiales	Tstip	421	6.5^-143^	1.9^-45^	2.2^-3^	1.3^-27^	9.1^-47^	217	1.0^-65^	6.2^-21^		5.8^-8^	1.3^-4^	9.5^-19^	YES
Onygenales	Mcani	417	2.0^-130^	7.2^-44^		1.6^-21^	4.9^-41^	208	1.1^-54^	2.7^-14^		1.5^-9^		2.4^-17^	YES
Onygenales	Pb-01	408	4.6^-136^	6.5^-46^		2.0^-23^	1.8^-45^	180	3.1^-57^	9.6^-16^		9.9^-9^	4.5^-8^	1.2^−11^	
Onygenales	Pb-03	410	1.7^−134^	2.4^−45^		2.0^−23^	1.9^−45^	180	1.6^−58^	1.7^−15^		3.5^−10^	4.5^−8^	1.2^−11^	
Onygenales	Abenh	384	1.1^−127^	2.5^−44^		2.1^−19^	3.3^−42^	222	3.9^−52^	2.0^−14^		1.5^−9^		1.4^−16^	YES
Onygenales	Aderm	424	1.5^−131^	3.9^−42^		3.8^−21^	1.2^−47^	201	1.6^−59^	1.4^−16^		1.6^−10^	3.7^−8^	2.5^−12^	YES
Onygenales	Tverr	409	5.0^−129^	2.6^−44^		8.1^−20^	3.7^−42^	208	2.8^−51^	1.8^−14^		9.1^−7^		2.3^−15^	YES
Onygenales	Ttons	429	1.6^−128^	1.0^−44^		1.8^−19^	1.9^−41^	207	2.1^−52^	1.8^−14^		1.3^−9^		3.6^−15^	YES
Onygenales	Mgyps	446	6.7^−133^	1.6^−46^		2.2^−20^	1.8^−42^	208	5.2^−52^	1.9^−14^		2.6^−8^		3.0^−16^	YES
Onygenales	Trubr	429	5.8^−129^	2.5^−44^		3.3^−20^	2.4^−42^	208	9.0^−52^	1.6^−14^		1.3^−9^		2.5^−14^	YES
Dothideomycetes	Chete	354	2.5^−86^	1.4^−34^		5.1^−15^	2.6^−27^	237	1.3^−43^	1.5^−14^		1.2^−10^		2.8^−14^	
Dothideomycetes	Ptrit	389	3.3^−85^	1.3^−34^		3.2^−15^	1.9^−26^	238	1.7^−49^	4.8^−14^		7.3^−12^		3.3^−15^	
Dothideomycetes	Snodo	405	2.4^−84^	6.7^−36^		3.6^−15^	2.9^−26^	242	2.0^−46^	2.1^−14^		3.3^−12^		4.6^−15^	
Dothideomycetes	Mgram	380	4.1^−73^	3.3^−34^		3.8^−13^	1.6^−20^	213	1.3^−47^	2.7^−16^		1.5^−10^	1.1^−4^	2.4^−8^	YES
Sordariomycetes	Tvire	358	2.3^−73^	2.4^−33^		5.7^−13^	1.6^−23^	269	9.2^−28^	7.3^−9^				1.7^−7^	YES
Sordariomycetes	Ndisc	422	2.0^−69^	3.8^−32^		1.0^−14^	4.0^−22^	1014	5.5^−20^	8.1^−9^			2.2^−4^	2.1^−8^	YES
Sordariomycetes	Tterr	427	6.0^−72^	1.8^−33^		2.2^−11^	8.0^−21^	524	5.2^−21^	6.6^−11^			8.6^−5^	3.9^−8^	
Sordariomycetes	Vdahl	382	1.1^−67^	1.6^−34^		8.^9−8^	2.5^−23^	383	2.0^−25^	8.9^−12^				4.9^−8^	
Sordariomycetes	Ncras	365	2.2^−68^	5.9^−32^		5.1^−14^	1.5^−22^	1001	6.0^−20^	2.0^−9^			2.1^−4^	2.5^−8^	YES
Sordariomycetes	Tatro	351	9.2^−75^	1.0^−33^		4.8^−13^	9.1^−24^	269	1.1^−28^	1.5^−9^				2.3^−10^	YES
Sordariomycetes	Ntetr	366	6.1^−69^	1.2^−32^		5.2^−14^	2.9^−22^	990	1.1^−21^	2.0^−9^			2.1^−4^	2.4^−8^	YES
Leotiomycetes	Sscle	386	2.9^−73^	8.4^−32^		1.1^−16^	2.1^−21^	249	2.9^−20^	1.1^−12^		5.4^−4^			
Sordariomycetes	Foxys	362	5.1^−67^	2.6^−31^		5.2^−12^	7.2^−22^	832	5.3^−18^	5.5^−10^					YES
Sordariomycetes	Fgram	361	2.5^−67^	3.0^−31^		4.8^−12^	4.1^−22^	836	1.3^−19^	6.2^−10^					YES
Sordariomycetes	Trees	367	1.9^−70^	1.0^−33^		8.5^−13^	6.2^−22^	274	2.9^−28^	4.2^−8^				1.4^−7^	YES
Sordariomycetes	Fvert	359	1.8^−67^	2.9^−31^		6.5^−12^	2.4^−22^	722	1.3^−18^	4.6^−10^					YES
Dothideomycetes	Mfiji	368	5.9^−70^	5.5^−33^		1.5^−11^	1.2^−19^	195	6.6^−47^	5.9^−16^		2.8^−11^	1.1^−4^	8.4^−13^	
Sordariomycetes	Moryz	380	5.2^−61^	6.5^−30^		8.2^−6^	3.5^−21^	294	4.3^−20^	6.7^−9^					YES

*YES indicates acidic region of FlbE is present. The nine orthologs used for motif discovery are in bold.

We then generated Hidden Markov Model (HMM) [45] profiles for the conserved motifs. Full length HMM profiles were also generated so that relative distances between the orthologs and the nine Eurotiales could be scored. The HMM profile scores are reported in Table 4.

### Full-length sequence comparison with HMM

When the sequences were evaluated using the respective full-length HMM profiles, all the FlbB and FlbE orthologs were scored at significant E values (Table 4). Among all the Eurotiomycetes, FlbE full-length HMM E values, in general, followed the trend of the respective FlbB E values (by which Table 4 is ordered). On the other hand, full-length HMM E values for the Sordariomycetes and Leotiomycetes FlbEs were higher, exhibited more interspecies variation and greater divergence from the FlbB scored order than those for the Eurotiomycetes. Additionally, there was much more variation in the length of the FlbE orthologs among the Sordariomycetes (e.g., 1014 residues for Ndisc compared to 201 for Anidu, Table 4). These findings suggested that there was substantial variation among the FlbE orthologs outside of the Eurotiomycetes (Table 4) at the species level. Note however, that all the FlbE orthologs had significant full-length HMM E values indicating that they encoded FlbE functionality.

### Motif conservation among orthologs

FlbB motifs B1, B3 and B4 were found at significant E values in all 40 FlbB orthologs (Table 4). That B1 and B4 are highly conserved is consistent with their being largely comprised of structural homologies. The fact that B3 is highly conserved suggests that it also encodes a region important for FlbB function.

B2, on the other hand, was limited to the nine Eurotiales from which the motifs were derived plus a lower confidence match with Tstip. This limited distribution, at first, would seem to be an artifact of the motif derivatization process since the motif was found to be highly conserved only in the orthologs that were used to define it. However, the three other conserved regions (B1, B3 and B4) had a wide distribution among the FlbB orthologs suggesting that conserved motifs extracted with our methodology were not always exclusive to the nine Eurotiales. Additionally, it is unlikely that insertions and deletions caused the limited distribution of B2 since both B2 and B3 include gapped regions in alignment of the 40 FlbB orthologs (Figure S1). One hypothesis for the limited distribution of B2 could be that it encodes a novel function that is not present in the other orthologs.

Motif distribution among the FlbE orthologs showed more variation than was seen in the FlbB motifs (Table 4, Figure 1 B). These differential and unique distributions could indicate that they encode various functionalities that arose at different points in the evolution of FlbE. While FlbE is not the primary focus of this study, the varying levels of motif conservation could be a means to prioritize areas for experimental investigation of FlbE function. Indeed, a portion of E1, the most conserved motif, has recently been shown to be essential for FlbE function [28]. Of particular interest for our study of FlbB functional determinants is that B2 and E2 share the same distribution. This shared distribution suggests that these two motifs may be functionally related.

### FlbB and FlbE function outside of Eurotiales

FlbB, FlbD and FlbE form a branch of the upstream conidiation pathway that has been well studied in Anidu and related fungi. All three of these proteins are crucial for timely conidiation with knockouts of the respective genes exhibiting a strong aconidial fluffy phenotype. The exact function the orthologs of FlbB and FlbE in other species is unknown, however, two orthologs of the FlbB/D/E pathway have been characterized in Ncras. The gross phenotype of a knockout of the FlbB ortholog in Ncras (NCU07379) was characterized by the Neurospora Genome Project (NGP) [46] with the results available on the Broad Institute website (http://www.broadinstitute.org/annotation/genome/neurospora/AlleleDetails.html?sp=S989&sp=S7000006085195119).This mutant conidiates normally. Unfortunately, the Ncras FlbE ortholog (NCU05255) has not been characterized by the NGP. However, a knock out of the third player in FlbB/D/E branch of the asexual development pathway, FlbD, has been shown to have no discernable phenotype by the Ebbole group [47] and to conidiate normally by the NGP (NCU01312). In view of this result, we hypothesize that a null allele of Ncras FlbE would also display a wild type phenotype in terms of conidiation.

## Discussion

The 40 FlbBs characterized in this study all contain highly conserved bZip and C-terminal Yap1-like domains, share the same general topology and are similarly sized. Additionally, all the orthologs have highly significant HMM E values for full length FlbB and conserved motif B1 and B4 profiles. These findings, combined with their unremarkable evolutionary relationships suggest that the FlbB proteins of the 40 species are indeed orthologs and likely function as key developmental regulators in a manner similar, but perhaps not identical, to Anidu FlbB (as noted above for Ncras). However, similarities and differences among the sequences identify both putative key functional determinants as well as important differences among the orthologs from the different orders and classes within the Pezizomycotina.

The eight Eurotiales that have an intact bZip DBD, Arg as the positive residue in the dimerization salt bridge, Glu in the 3^rd^ heptad ‘d’ position, all six relevant conserved cysteines and the B2/E2 conserved motif pair (Figure 2, yellow highlight) are likely to share functionality with Anidu FlbB to the extent that results from experimental characterizations of that protein could apply to each. Additional support for shared functionality is provided by the low E values obtained by all eight orthologs (<8^-230^) when evaluated with the nine Eurotiales full length HMM profile (Table 4). Follow up experimental work in this regard could involve cross-complementation studies among these eight species. For example, it was recently shown that the Anidu FlbB sequence was able to partially complement an Afumi FlbB^-^ strain [48]. Such experiments could be informative as to the determinants of particular functions. Cross-complementation studies among fungal proteins have proven to be informative and an effective way to transfer characterizations of proteins in one species to other, less genetically amenable, species [47]-[49], [50], [51].

However, FlbB orthologs outside Eurotiales likely function differently than those most closely related to Anidu. Differences in function could be due to: alterations in the bZip dimerization domain, differential distribution of conserved cysteines and the lack of the putative interaction motif pair B2-E2. For example, the H19 bZip DBD is present in all the FlbB orthologs but the dimerization profiles of the 22 Eurotiomycetes likely differ from the other 18 orthologs because the charge along the hydrophobic bZip dimerization interface differs in polarity and location in the two groups.

The presence of a histidine (Anidu His93) in the DBD, which we designated H19, puts FlbB DBDs outside of any of the described bZip subfamilies [35]–[37]. The H19 DBD has remarkably limited distribution, especially considering the ubiquitous nature of bZip TFs in Eukaryotes. Furthermore, of the 22 putative bZips found in Anidu [52], only FlbB contains a H19 DBD (data not shown). These two conservation profiles suggest that H19 plays a specific role in FlbB function.

Although DBD position 19 is among the five residues in contact with DNA, there is evidence that it does not always participate in DNA recognition [37]. Residues found in position 19 of bZip DBDs include cysteines (C19), serine (S19), tyrosine (Y19) and phenylalanine (F19) [37]. It has been shown that serine and cysteine in position 19 do not necessarily contribute to DNA binding [53], [54]. On the other hand, phenylalanine in position 19 has been shown to be important in fungi for Pap1 recognition sequence binding [35]. Contrary to this finding, the H19 containing Anidu FlbB has been demonstrated to bind these same Pap1 sites [14], [35]. This apparently unaltered binding site specificity of a F19->H19 substitution supports the relative unimportance of position 19 for DNA binding. These differential findings may have arisen because the functional difference between F19 and H19 has more to do with relative affinity rather than absolute site recognition. This could be investigated using reciprocally cross-mutated versions of FlbB and Pap1 or by analyzing the effect of a Phe->His substitution in FlbB.

In addition to DNA binding site recognition, phosphorylation of S19 [54], [55] and oxidation of C19 [53], [56] have been shown to be post translational mechanisms for abrogating DNA binding. A clue as to the functional importance of the histidine in position 19 could be that that histidine, too, can be phosphorylated [57]. This possibility is highly speculative as histidine phosphorylation occurs in only a few specific functions such as phosphorylations in the first step in two-component system signaling, of non-adenine nucleoside diphosphate by nucleoside diphosphate kinase and of histone H4 by histidine kinase [58]. Another mechanism by which the DNA binding activity of the H19 DBD could be modulated is that histidine, with a pI of 7.6, could conceivably function as a pH sensor such that small changes in nuclear pH could modulate DNA binding. Although there are no detailed studies on the pH of fungal nuclei, it is known that the cytoplasmic pH of Anidu is maintained at 7.6 under a variety of external conditions [59]. In such an environment, relatively small shifts in pH will alter the charge of histidine and thereby alter its affinity for DNA. To date, no link has been described between FlbB and pH response or with the regulator of this response in Anidu, PacC [60], however, the possibility that DNA binding could be modulated through one or both of these mechanisms makes experimental work to confirm or refute these hypotheses quite compelling.

Experimental results show that Anidu FlbB G70 residue is not essential for target DNA recognition in vitro but seems to be necessary to increase the efficiency of the binding. Based on this observation, we could suggest that although the consensus (NxxAQxxHR) sequence defines the specificity of the target DNA binding, auxiliary residues, as G70 in the case of FlbB, modulate the efficiency of the interaction. For example, a Gly residue plays a key role in the formation of the Hap complex in Anidu [61]. It is part of a region necessary for the recruitment of HapX to the Hap complex and in subsequent binding to the regulatory sequence [62]. As the region containing G70 is conserved in all 40 orthologs, any specific knowledge gained from the study of any of the 40 FlbB orthologs in this respect would likely apply to all of them.

The similarities between the FlbB orthologs and the C-terminal region of Yap1 and Pap1 raise two questions. First, although the C-terminal regions of the FlbB orthologs likely function similarly to these two oxidative response regulators (i.e., mechanistic homology) is FlbB, in fact, a functional homolog? That is unlikely as the regulatory role of FlbB is directed towards the induction and control of cellular development [13]-[15] rather than specific responses to oxidative stress. Additionally, NapA, a Yap1/Pap1 functional homolog has been characterized in Anidu [63]. This protein, besides not being linked to conidiation, has been shown to function as an oxidative response regulator and shares the signature phenylalanine-containing PAP subfamily bZip DBD (F19) [35] with Yap1 and Pap1 rather than the distinctive H19 DBD of FlbB [63]. The second question is whether or not this region participates in the modulation of localization and stability of FlbB as it does in Yap1 and Pap1. Such modulation is consistent with the observed alterations in Andiu FlbB nuclear localization at different growth and development stages [14], however, further experimental work is needed to establish links between this proposed mechanism and FlbB localization.

The distribution of cysteines in the FlbB orthologs provides little information in terms of determining functional cysteine pairs except for two points: 1) the eight orthologs containing all six highly conserved cysteines (Figure 2, Figure 4, Figure S1) could function similarly to Anidu FlbB in that the same options for intramolecular disulfide bond(s) formation would exist, and 2) a single disulfide bond, such as could be formed between single cysteines pair C272 and C382 in the Dothideomycetes could be sufficient for minimal functionality required for a hypothetical redox control mechanism (at this point, a putative requirement for FlbB function). In this regard, it is worth noting that Yap1 and Pap1 form different disulfide bonds depending on conditions and/or to alter the longevity of the response [43], [44] but also that a single cysteine pair could be sufficient for function of this mechanism although in this case there would be only one level of response.

Support for C272 being the most likely cysteine to form a di-sulfide bond with C382 comes from the findings that 1) it is the second most conserved cysteine in FlbB among the orthologs, 2) it is within the highly conserved B3 motif and a region important for FlbB function [15], and 3) the area of B3 that contains C272 contains predicted order and helix secondary structure which is consistent with the possibility of structure formation with C-terminal region in a manner similar to Yap1 [17].

It should also be noted that both Yap1 and Pap1 contain functional nuclear localization sequences (NLS) [64], [65] and nuclear export signals (NES) [66], [67] that function in conjunction with di-sulfide bond-mediated NES masking in order to affect differential nuclear/cytoplasmic localization. Neither NLS nor NES, both of which can be cryptic [68], [69], have been identified in any of the FlbB orthologs but experiments to elucidate them would be worth pursuing as a means of further understanding the Yap1/Pap1 mechanistic connection.

Interpretation of the order predictions relative to the conserved regions suggests that both proteins contain a linker separating regions of structure. In FlbB, the proposed linker lies in the less conserved region that contains few structure predictions between motif B2 and B3 (Figure 1 A). In FlbE, a region with the same characteristics lies between motifs E4 and E5 (Figure 1 B). The majority of conserved motifs in both proteins are largely associated with predictions of order (B1, B3, B4, E1, E4 and E5). Motifs B2, E2 and E3 have less structure predictions associated with them suggesting that some of the conservation in these regions is not related to structure.

The limited distribution of certain motifs (B2, E2, E3, E4, E5 and the FlbE acidic region) suggests that they may encode functional aspects that are not present in all the orthologs. Furthermore the *shared* limited distribution of conserved motifs B2 and E2 suggests that they may be linked functionally. Since FlbB and FlbE have been shown to interact in vivo and in vitro [13], of particular interest here is the possibility that B2 and E3 may facilitate this interaction. While highly speculative, this hypothesis specifies a starting point for mutational studies towards this end.

### Conclusions

*In silico* analyses of the FlbB orthologs from 40 closely related filamentous fungi have revealed similarities and differences at the domain, motif and residue level. The 40 FlbB orthologs are highly similar and likely function as key developmental regulators in a manner similar to Anidu FlbB. While all contain structural homologies to bZip and the C-terminal region of Yap1, differences in key residues differentiate some orthologs from others. Changes in the bZip dimerization domain affect specificity and affinity for dimerization partners and could thereby alter the transcriptional activation profile of the functional dimers. The presence or absence of the conserved motif B2 and E2 pair could influence FlbB/FlbE interactions. Differences in the C-terminal cysteine pattern may provide a means for increased and/or differential functionality. Eight species: Anidu, Aterr, Anige, Aflav, Aoryz, Aclav, Afumi and Nfisc contain all the Anidu-centric features identified in this study and would therefore likely be able to functionally complement one another. However, the remaining orthologs lacked one or more of these critical features and could therefore function differently. Indeed, experiments have shown that Ncras FlbB apparently does not play a role in conidiation. However, its role in other cellular processes has not been investigated. Nevertheless, the differentially conserved residues and motifs identified here comprise a list of targets for functionally-directed mutational studies.

### Future directions

The abundance of fully sequenced fungal genomes makes in-depth approaches such as presented here feasible for almost any fungal protein. Indeed, more than 100 sequenced genomes are now available with more forthcoming in the near future from efforts like Joint Genome Institute (JGI) and the Fungal Genome Initiative (FGI). This study was focused on Anidu but the approach lends itself to the study of almost any fungal protein. Our Anidu-centric focus was intentional since we were interested in exploring the distribution of the experimentally characterized functionality of that species. Conducting a similar analysis based on Ncras, for example, could yield information about functional features encoded in that species that may not necessarily be present in Anidu. In some cases, this type of analysis could be informative as to the origin of proteins that have arisen through recombination or have been adapted to ‘new’ functions [70]. Such analyses can be used to augment traditional approaches prior to initiating laboratory experiments but can also elucidate details of protein function and potentially lead to information regarding the origin of proteins and motifs that serve particular functions, protein interrelationships and/or pathway evolution.

## Methods

### Verification of ORF and exon/inton calling

Genomic sequences were manually compared with protein sequences in order to verify gene finding results reported by sequencing projects. When possible, experimentally verified protein sequences were used for reference (i.e., in Anidu and Afumi, both proteins have been sequenced). Phylogenetic relationships in terms of homology and splice site conservation among the orthologs were the main criteria to support the existence or absence of introns. Results from TBLASTN, BLASTX, Genewise (http://www.ebi.ac.uk/Tools/Wise2/) and Fgenesh (Softberry) [71] were considered along with manual verification of splice sites.

### Shannon entropy calculations

Following alignment with CLUSTAL, column entropy [72] was calculated using the entropy web server http://www.hiv.lanl.gov/content/sequence/entropy /entropy_one.html. Amino acid substitutions were not allowed in the calculation. Data for columns in which Anidu contained a gap were not considered for conserved motif selection. Columns in which the five-residue moving average of entropy was less than 0.5 were considered to have significant levels of conservation and were candidates for inclusion in motifs.

### Structure predictions and homology searches

Order was predicted using VLS2B (http://www.ist.temple.edu/disprot/Predictors.html) [73]. GORIV was used for secondary structure prediction (http://npsa-pbil.ibcp.fr/cgi-bin/npsa_automat.pl?page=npsa_gor4.html) [74]. NCBI Conserved Domain Database search [75] results were taken from BLAST for Anidu FlbB. The 40 FlbB orthologs were scored for the presence of conserved domains by directly accessing the SMART database [30] through the website interface (http://smart.embl-heidelberg.de/). Conserved and domain search results were considered significant if the E value was less than 0.01. The Homstrad database [76] was queried using the Fugue sequence-structure homology recognition server [18] (http://tardis.nibio.go.jp/fugue/prfsearch.html) using default settings to evaluate the FlbB orthologs for structural homologies. Searches of NCBI non-redundant database and recently completed genomes from the FGI and JGI were searched for H19 bZip DBD signatures using HMMs with profiles generated from the DBD of 40 FlbB orthologs and, in a recursive manner, profiles generated from extant H19 bZip DBD sequences found outside Pezizomycotina. Both standalone [72] and web-based (http://mobyle.pasteur.fr/cgi-bin/portal.py) HMM programs were used.

### Evaluation of the acidic region of FlbE

The presence or absence of the C-terminal acidic region in FlbE orthologs was determined by using the Grantham method [77] as implemented on the ExPASy website (http://www.expasy.org/tools/protscale.html) [78] using a window of 15 residues. The acidic region was deemed to be present if the moving average of polarity scores exceeded 10 for 10 consecutive residues. An additional constraint was that this acidic region needed to be adjacent to the C-terminal end of motif E5.

### Generation of the FlbB C382A mutant strain

A pair of complementary oligonucleotides, flbB-C382A+1 and flbB-C382A-1, was designed bearing a TGC (coding for Cys382)-GCA (coding for an Ala382) substitution (Table 5). These, plus two oligonucleotides flanking the flbB locus (flbB-PP1 and flbB-GSP4) were used to generate products for a fusion-PCR mutation procedure. Briefly, using genomic DNA from a strain expressing FlbB::GFP::pyrG [15] as a template, two DNA fragments were amplified: one of 2.9Kb covering the *flbB* promoter plus the corresponding sequence of the *flbB* locus (oligonucleotides flbB-PP1 and flbB-C382A-1) and the second one of 3.5Kb covering the rest of *flbB* locus, *gfp*, *pyrG* and the 3′ untranslated region (oligonucleotides flbB-C382A+1 and flbB-GSP4). Both fragments were fused [79], purified and used to transform the wild type strain TN02A3 [80].

**Table 5:** Oligonucleotides used to generate the FlbB-C382A allele.

Designation	Sequence (5′- 3′)
flbB-PP1	GTTTTCTGGTCCTCGGTCAACCGGTGG
flbB-GSP4	GAAAGGTGCGTGGGTTCGAATCCCACC
flbB-GSP2	TGAATACATCGTCTCATCAGCATGCCGGGT
flbB-C382A+1	GAGAACAAGGTGCGC**GCA**TACGGATTCGGG
flbB-C382A-1	CCCGAATCCGTA**TGC**GCGCACCTTGTTCTC
flbB-sek5	GCCGGGAAAACGCAACGC

The substituted codon is in bold underlined font.

The parental strain allowed homologous recombination events either upstream or downstream of the TGC->GCA substitution, and this was reflected at the phenotypic level with transformants showing both wild type and aconidial phenotypes. Transformants were checked by Southern-blotting to confirm the appropriate recombination (data not shown) and the presence of the TGC->GCA substitution was confirmed by sequencing. With this aim, a 3.1Kb amplicon covering the *flbB* promoter and the entire coding region was generated using oligonucleotides flbB-PP1/flbB-GSP2, and sequenced using oligonucleotide flbB-sek5.

The phenotype of the strain expressing the FlbB allele bearing the C382A substitution was analysed in *Aspergillus* Minimal Media [81] after 72 hours of growth and compared with the parental wild type TN02A3, Δ*flbB*, *flbB100* (G70R point substitution) and *flbB102* mutant strains, the latter being a mutant allele with a truncation after amino acid P305 [15].

## Supporting Information

Figure S1 **Alignment of the 40 Pezizomycotina FlbB orthologs used in this study.** Anidu FlbB is in bold. Motifs B1, B2, B3 and B4 are labeled and highlighted in green or yellow in the nine sequences that were used to generate the motifs. The five signature residues of the bZip DNA binding domain are highlighted in purple. The first four heptads of the bZip dimerization domain are identified by brackets with the residue positions labeled a – g according to convention (only the first residue of the fourth heptad was positively identified). In the bZip dimerization domain, hydrophobic and charged residues in positions ‘a’ and ‘d’ (zipper forming residues) are highlighted in grey or orange, respectively, and salt bridge residues are highlighted in light blue. Specific residues discussed in text (Anidu numbering) are labeled above the alignment. Cysteine residues are additionally highlighted in light red. Residues flanking intron locations are in bold italic.
(PDF)

Figure S2 **Alignment of the 40 Pezizomycotina FlbE orthologs used in this study.** Anidu FlbE is in bold. Conserved motifs E1, E2, E3, E4 and E5 are labeled and highlighted in green or yellow. The acidic region is highlighted in purple. Residues flanking intron locations are in bold italic.
(PDF)
