# Supplementary material for: Elucidation of Functional Markers from Aspergillus nidulans Developmental Regulator FlbB and Their Phylogenetic Distribution
Source: PLoS One. 2011 Mar 10;6(3):e17505. doi: 10.1371/journal.pone.0017505 (PMC3053368; doi:10.1371/journal.pone.0017505)
Supplement: Figure S1 — Alignment of the 40 Pezizomycotina FlbB orthologs used in this study. Anidu FlbB is in bold. Motifs B1, B2, B3 and B4 are labeled and highlighted in green or yellow in the nine sequences that were used to generate the motifs. The five signature residues of the bZip DNA binding domain are highlighted in purple. The first four heptads of the bZip dimerization domain are identified by brackets with the residue positions labeled a – g according to convention (only the first residue of the fourth heptad was positively identified). In the bZip dimerization domain, hydrophobic and charged residues in positions ‘a’ and ‘d’ (zipper forming residues) are highlighted in grey or orange, respectively, and salt bridge residues are highlighted in light blue. Specific residues discussed in text (Anidu numbering) are labeled above the alignment. Cysteine residues are additionally highlighted in light red. Residues flanking intron locations are in bold italic. (PDF) [file pone.0017505.s001.pdf]

```
Trees      ----------HCFGASRAAMSSSLSPDH-AEGSTSRASTEGTDKGVLSS----------- 38
Tvire      ------------FEASRAAMSSSLSPDH-AEGGTSRASTEGTDKGVLSS----------- 36
Tatro      --------------------MSSLSPDQ-IEGGASQASTEGTDKGVLSS----------- 28
Vdahl      -------------------MASSLPSDQQGSASHARSSSAEQDNGPLSN----------- 30
Moryz      --------------------MQSYEGS--------GGSDGGHDKSPLSS----------- 21
Tterr      --------------------MASVSSNQPSQRSFGGGSSTTQDRPLLST----------- 29
Ncras      --------------------MTSSLPLQP-LATYAEDPHSGPDSNPLST----------- 28
Ntetr      --------------------MTSSLPLQP-LATYAEDPHSGPDSNPLST----------- 28
Ndisc      ---------------------MASLSFQP-LAPYAEDPHAGSDSNPLST----------- 27
Foxys      --------------------MAAFNANLAGATERSFSSSLSPDRVVASSE---------- 30
Fvert      --------------------MAAFNANLAGATERSFSSSLSPDRVVASSE---------- 30
Fgram      --------------------MSVFNNHLAGATERSFSSSLSPDRIIASSE---------- 30
Sscle      --------------------MTSPSPENFDYTNRSDRGDYASEQSEGAR----------- 29
Ptrit      --------------MAPTYPPPGAVNEYPSPAASRRPSECFSEMSMDSSRRGSLAGFASD 46
Snodo      MSHNRYMSWSEGLTMGPVYPPPGAGADYPSPAASRRASECFSDMTH--SRRGSLAGFASE 58
Chete      ------------------------------MAATIKTDPSVGDSSH-------------- 16
Mfiji      -------------------MAAYTAGAHPMPHALPQPDQMRHDVSYD------------- 28
Mgram      -------------------MTTFHA-AH-QPHGLSMSDGMSHDVGYDT------------ 27
Abenh      ------------------------------------------------------------
Tverr      ---------------------------------------------MG------------- 2
Trubr      -----------------------MASGAPQASPDANS--VPTGIDPG------------- 22
Ttons      -----------------------MASGAPQASPDANS--VPTGIDPG------------- 22
Mgyps      -----------------------MASSAPQSSPEANR--VPTGIEPAPELIRSCGGNKSA 35
Mcani      ---------------------------------MGGN--YPASINLGP------------ 13
Cimmi      -----------------------MEPSGTNQGIMAGQSGVPVTQTLSSNVGSAPG----- 32
Cposa      -----------------------MEPSGTNQGIMAGQSGVPVTQTFSSNVGSAPG----- 32
Pb-01      -----------------------MASFGQYGGLASDK----------------------- 14
Pb-03      -----------------------MASFGQYGGLASDK----------------------- 14
Aderm      -----------------------MASLGHHNGSPSNQ----------------------- 14
Afumi      -----------------------MASMNGGVMPVDY---GHTR----------------- 17
Nfisc      ------------------------------------------------------------
Aclav      ----------------------------------------MYT----------------- 3
Aflav      -----------------------MASLNEGVLPLEA---DALNRQNDE------------ 22
Aoryz      -----------------------MASLNEGVLPLEA---DALNRQNDE------------ 22
Anige      -----------------------MTSINGGPMSVDL---SAANRQTMT------------ 22
Aterr      -------------------MTAAMATMNGGVMPMDH---GTIDFNTLNG----------- 27
Anidu      -----------------------MTSISSRPIPLDLNAMDSLNRQSLAQ----------- 26
Pchry      ------------------------------------------------------------
Pmarn      -----------------------MSGYPSGGHTG--GMGYNDPYAQYGAISMFRL----- 30
Tstip      -----------------------MSSYPSGNQAAGMGIGYNDQFAR-------------- 23
```

B1

H93

```
Trees      ------------------LNLNFFKSLSD------------KKVTRTGN--PPKRRGPKP 66
Tvire      ------------------LNLNFFKNLSD------------KKVTRTGN--PPKRRGPKP 64
Tatro      ------------------LNLNFFKNLSD------------KKVTRTGN--PPKRRGPKP 56
Vdahl      ------------------LNLGFLKGLTE------------KKATRDGQ--PPKRRGPKP 58
Moryz      ------------------IPSNFLRNLTD------------KRTKSDAP--APKRRGPKP 49
Tterr      ------------------MNLDILRTLTE------------KRTTRDGQ--PQKRRGPKP 57
Ncras      ------------------LNLTILKSLSD------------KK-TRDGQ--TPKRRGPKP 55
Ntetr      ------------------LNLTILKSLSD------------KK-TRDGQ--PPKRRGPKP 55
Ndisc      ------------------LNFTILQNLAD------------KK-TRDGQ--APKRRGPKP 54
Foxys      ----------DDNTAASALDPDFYKSLTD------------KRTTRDGN--PPKKRGPKP 66
Fvert      ----------DDNAAASTLDPDFFKSLTD------------KRTTRDGN--PPKKRGPKP 66
Fgram      ----------DDNAAA--LDPEIYKSLAD------------KRTTRDGN--PPKKRGPKP 64
Sscle      --------------SPLSMSLGFLKTLTER-----------KSTRVDGS--TPKRRGPKP 62
Ptrit      MESAGQWQSLGSDTKSPLAQFGFFKSLT-D-----------KKTTRDGQ--PPKRRGPKP 92
Snodo      MESTGQWQSIGSESKSPLAQFGFFKGLT-E-----------KKTTRDGQ--PAKRRGPKP 104
Chete      ------FNGSGSESKSPLAQFGFFKSLT-D-----------KKTTRDGQ--PPKRRGPKP 56
Mfiji      -----------------------------------------------GQ--PPKRRGPKP 39
Mgram      -------NPPGSD-RSPLHDLGFYKNITPE-----------QKQTKDGQ--PAKRRGPKP 66
Abenh      ------------------------------M----------KKSPSDGQQGQPKRRGPKP 20
Tverr      ------PPIVPI-KKPSLANLPFIRNFSDPL----------KKSPSDGQQGQPKRRGPKP 45
Trubr      ------PPIVPI-KKPSLANLPFIRNFSDPL----------KKLPNDGQQGQPKRRGPKP 65
Ttons      ------PPIVPI-KKPSLANLPFIRNFSDPL----------KKLPSDGQQGQPKRRGPKP 65
Mgyps      SACIGPPPTVPK-KNPSLVNLAFIKNLSDPV----------KKIPSDGQ--QPKRRGPKP 82
Mcani      ------PPTVPK-KNPSLVNMSFLKNFNDPL----------KKV-TDEQRQQPKRRGPKP 55
Cimmi      ------DMTAPVSAESSFISMPFLKALGGGL----------QKKTRDGQ--PPKRRGPKP 74
Cposa      ------DMTAPVSAESSFISMPFLKALGGGL----------QKKTRDGQ--PPKRRGPKP 74
Pb-01      ---------------NKKIENVDVDVDVDDF----------EKTTTRGQ--PQKRRGPKP 47
Pb-03      ---------------NTKNENADVDVDVDD----------------VGQ--PQKRRGPKP 41
Aderm      ---------------SSRNGNAG-----DQ----------------EGQ--PPKRRGPKP 36
Afumi      -----GAPSQTSLKTPTSMSTNFFQFFGG-----------QKKVTRDGQ--PAKRRGPKP 59
Nfisc      ------------------MSTNFFQFFGG-----------QKKVTRDGQ--PAKRRGPKP 29
Aclav      -----GAPSQTSIKTPTSMGANFFQFFAG-----------QKKVTRDGQ--PAKRRGPKP 45
Aflav      ---LNGRQSQGSLMK-ASMGTEWFRFFGSG----------QKKVTRDGQ--PAKRRGPKP 66
Aoryz      ---LNGRQSQGSLMK-ASMGTEWFRFFGSG----------QKKVTRDGQ--PAKRRGPKP 66
Anige      ---PEAMPSQPNSRP-STIGADFFKFFSGT----------QKKVTRDGQ--PAKRRGPKP 66
Aterr      ---TNDRQSQSSLK-----GADWFKFFGGG----------QKKVTRDGQ--PAKRRGPKP 67
Anidu      ---SASPAGQDTPGMRGSISTDFFKFFGAGGQ--------AKKTTRDGQ--PAKRRGPKP 73
Pchry      ------MVEEPQTPSSKGFGAEIFKIFGGGGSGSAASANGPKKATRDGQ--QPKRRGPKP 52
Pmarn      ---LQENAPMYAPAPEPNKFNGFLKALGAG----------TKKTKGDGQ--PAKRRGPKP 75
Tstip      --------QYEAPAPEQTKFNGFLKALGAG----------TKKTKGDGQ--PPKRRGPKP 63
                                                                *:*****
```

B1

1 2 3 4

H93 abcdefgabcdefgabcdefga

```
Trees     DSKPALTRRQELNRQAQRTHRERKELYIKALEDEVLRLKEVFSTISQDK----------- 115
Tvire     DSKPALTRRQELNRQAQRTHRERKELYIKALEDEVLRLKEVFSTISMDK----------- 113
Tatro     DSKPALTRRQELNRQAQRTHRERKEIYIKALEDEVIRLKEVFSTMSLDK----------- 105
Vdahl     DSKPAMTRRQELNRQAQRTHRERKELYIKALEDEVLRLKEVFSNMSQDK----------- 107
Moryz     DSKPALTRRQELNRQAQRTHRERKELYIKALEDEVLRLKETYSNVSQAK----------- 98
Tterr     DSKPALTRRQELNRQAQRTHRERKELYIRALEDEVLRLKEIFSHVSQDK----------- 106
Ncras     DSKPALTRRQELNRQAQRTHRERKELYIKALEDEVLRLKEIFGTISQDK----------- 104
Ntetr     DSKPALTRRQELNRQAQRTHRERKELYIKALEDEVLRLKEIFGTISQDK----------- 104
Ndisc     DSKPALTRRQELNRQAQRTHRERKELYIKALEDEVLRLKEIFSTISQDK----------- 103
Foxys     NSKPALTRRQELNRQAQRTHRERKELYIKALEDEVLRLKEVFSNVSLDR----------- 115
Fvert     NSKPALTRRQELNRQAQRTHRERKELYIKALEDEVLRLKEVFSNVSLDR----------- 115
Fgram     NSKPALTRRQELNRQAQRTHRERKELYIKALEDEVLRLKEVFSNVSLDR----------- 113
Sscle     DSKPALTRRQELNRQAQRTHRERKELYIKALEQEVLRLKENFSNVTQSK----------- 111
Ptrit     DSKPALTRRQELNRQAQRTHRERKELYIKALEQEVIRLKDTFATTSRER----------- 141
Snodo     DSKPALTRRQELNRQAQRTHRERKEMYIKALEQEVLRLKETFASTTRER----------- 153
Chete     DSKPALTRRQELNRQAQRTHRERKELYIKALEQEVIRLKDTFAATSRER----------- 105
Mfiji     DSKPALTRRQELNRQAQRTHRERKEMYIKALEQEVLRLKEMFAQSERER----------- 88
Mgram     DSKPALTRRQELNRQAQRTHRERKEMYIKALEQEVLRLKETFSQTTRER----------- 115
Abenh     DSKPAQTRRQELNRQAQRTHRERKEQYIRALEVEITRLREGYATDIEGA-------NVSI 73
Tverr     DSKPAQTRRQELNRQAQRTHRERKEQYIRALEVEITRLREGYATDIEGA-------NVSI 98
Trubr     DSKPAQTRRQELNRQAQRTHRERKEQYIRALEVEITRLREGYATDIEGA-------NVSI 118
Ttons     DSKPAQTRRQELNRQAQRTHRERKEQYIRALEVEITRLREGYTTGIEGA-------NVSI 118
Mgyps     DSKPAQTRRQELNRQAQRTHRERKEQYIRALEVEITRLREGYANDMESA-------NMSI 135
Mcani     DSKPAQTRRQELNRQAQRTHRERKEQYIRALEVEISRLREGYASDIEGA-------NMSI 108
Cimmi     DSKPAQTRRQELNRQAQRTHRERKEQYIRALEIEISRLREGYANDINSA-------NMSL 127
Cposa     DSKPAQTRRQELNRQAQRTHRERKEQYIRALEIEISRLREGYANDINSA-------NMSL 127
Pb-01     DSKPAQTRRQELNRQAQRTHRERKEQYIRALETEVSRLRECYSNDMSTT-------NLSM 100
Pb-03     DSKPAQTRRQELNRQAQRTHRERKEQYIRALETEVSRLRECYSNDMSTT-------NLSM 94
Aderm     DSKPAQTRRQELNRQAQRTHRERKEQYIRALETEISRLRECYSNDMSASNMSIQASNMSI 96
Afumi     DSRPALTRRQELNRQAQRTHRERKEQYIRSLEIEVSRLREAFTQEMSAA-------NLAV 112
Nfisc     DSRPALTRRQELNRQAQRTHRERKEQYIRSLETEVSRLREAFTQEMSAA-------NLAV 82
Aclav     DAKPAMTRRQELNRQAQRTHRERKEQYIRSLETEVSRLREAFTQEMSAA-------NLAV 98
Aflav     DSKPALTRRQELNRQAQRTHRERKEQYMRALETEVSRLREAYTQEISAA-------NLTV 119
Aoryz     DSKPALTRRQELNRQAQRTHRERKEQYMRALETEVSRLREAYTQEISAA-------NLTV 119
Anige     DSKPALTRRQELNRQAQRTHRERKEQYIRALETEVSRLREAYTQEISAA-------NLTV 119
Aterr     DSKPALTRRQELNRQAQRTHRERKEQYMRSLETEVSRLREAYTHEISAA-------NLTV 120
Anidu     DSKPALTRRQELNRQAQRTHRERKEQYIRALESEVARLREAYTQEISAA-------NLAV 126
Pchry     DSKPALTRRQELNRQAQRTHRERKEQYMRALETEVSRLREAYTTEIAEA-------NTSI 105
Pmarn     DSKPALTRRQELNRQAQRTHRERKEQYIRALESELSRLREVYSVDINTS-------NQKI 128
Tstip     DSKPALTRRQELNRQAQRTHRERKEQYIRSLESELSRLREVYSVDINAA-------NQKI 116
          :::** ******************* *:::** *: **:: :
```

B1 → ← B2

```
Trees     -------QKLFDENKQLKEILAQR----GIQLPRVGGV-DESSSVTQPMSVSAS--GNSF 161
Tvire     -------QKLVDENKQLKEVLVQR----GIQLPRID---DSGSSAAQPMSVSAS--GNSF 157
Tatro     -------QKLYDENKQLKEVLAQR----GIQLPKLSSVDDSGISATQAMSVSAS--GNSF 152
Vdahl     -------DKVAEENKQLRQLLTQH----GIPLSSVG-LDDTVSNPSGGYTASASPSGYSH 155
Moryz     -------DNLSNENRQLKQLLAQNGISFGGAMGIASG--MMDDGSIDYASSSAT--GSGY 147
Tterr     -------ERLAEENRQLKAILAQNGLGVGALIGRGGGGGGVGDDSVSNPSVGYS--PSSS 157
Ncras     -------ERLAEENRRLKAKLAQN-DAAGDPIAADTNFFDQMVSSPSGPSPGYT--SSSS 154
Ntetr     -------ERLAEENRRLKAKLAQN-DAAGDPIAADTNFFDQMVSSPSGPSPGYT--SSSS 154
Ndisc     -------ERLAEENQRLKAKLAHN-DAAGDPIAADTNFFDQMVSSPSGPSPGYT--SSSS 153
Foxys     -------ERLADENKRLRDTLAQA----GLQHSSPHVAGSATSRGPDSANTSSPPLTSRS 164
Fvert     -------ERLADENKRLRDTLAQA----GLQHSSPHVAGSATSG---SVNTSSPPLTSRS 161
Fgram     -------ERLADENKRLRDTLVQA----GLQHSSPHVAGSATSRGFDSNNTGSSPLTSQS 162
Sscle     -------QSLAEENRQLKQLLAQH----GIQWDGSGGFDDFTNTQSLGFRSSDS--PDSY 158
Ptrit     -------DAFAEENRRLRELLMAHGISFEHMTSPTNGMGQVGSSTYGSSN-GSISG-YGP 192
Snodo     -------DAFAEENRRLKELLMAHGIAFDLANPPNNGMPHVSSSTYGSSN-GSVSG-YGA 204
Chete     -------DAYAEENRRLRELLMAHGIAFDLQGRPSNGMAQVGSSTYGSST-GSVSG-YGP 156
Mfiji     -------NAFAEENRRLKELLMQHGISYDFAASPIKFTRENSGGGYGASSSGSISGSYRG 141
Mgram     -------DTIAEENRRLKELLQQHGISYDFGSSPIRFKHENSS--YGPSSSGSISGSYRG 166
Abenh     MQQKQALEELRDENRMLKEILASHGINFEAELEKRGAPVQST----PQESSYGGSVAPSL 129
Tverr     MQQKQALEELRDENRMLKEILASHGINFEAELEKRGAPVQST----PQESSYGGSVAPSL 154
Trubr     MQQKQALEELRDENRMLKEILASHGINFEAELEKRGAPVQST----PQESSYGGSVAPSL 174
Ttons     MQQKQALEELRDENRMLKEILASHGINFEAELERRGAPVQST----PQESSYGGSVAPSL 174
Mgyps     MQQKQTLEEVKEENRILKEILASHGINFEAELERRSAPVQST----PQESSYGGSISASH 191
Mcani     MQQRQNLEELKEENRVLKEVLACHGINVEAELERRKIAIQNT----PQESSYGGSTT--- 161
Cimmi     HQQRQTLESQKEENRILREILLSHGFNVDAELEQRRGMTRAG----SQRS-YQESSAQSH 182
Cposa     HQQRQTLESQKEENRILREILLSHGFNVDAELEQRRGMTRAG----SQRS-YQESSAQSH 182
Pb-01     QQQKKILQEQKDENAMLRQLLTAHGVPFEAELERRKASLRSGN-NRAYTSALVGRTSQAH 159
Pb-03     QQQKKILQEQKDENAMLRQLLTAHGVPFEAELERRRASLRSGN-NRAYTSASVGRTTQAH 153
Aderm     QQQRRVLQEHRDENILLKQILASHGIPFEAELEQRKLALRSGA-RHAHTDSFAGSTAHSH 155
Afumi     VQHREMLQTVNDENAILKELLTAHGIQFEAELERRRAERRSAG-RGFQSSPL--AGSSVV 169
Nfisc     VQHREMLQTVNDENAILKELLTAHGIQFEADLERRRAERRSAG-RGFQSSPL--AGSSVV 139
Aclav     VQHRDMIQAVNEENAFLKEILVAHGIQYEPELERRRAERP----RFQQSSPF--ASSSVA 152
Aflav     HQHREMVRSLSEENNILKEILAAHGISYEAEVERRKAERTSTTNATYQSSPF--ASSSVG 177
Aoryz     HQHREMVRSLSEENNILKEILAAHGISYGAEVERRKAERTSPTNATYQSSPF--ASSSVG 177
Anige     VQHRDMVQSLSDENAILKDILTAHGINYEPEVERRKAERPTIG---YQSSPF--ASSSTG 174
Aterr     HQHREMIQSLSDENRILKEILAAHGIQFEPELERRKAERPHPG--YHQSSPF--ASSSVA 176
Anidu     HQHREMANSISEENNMLKEILVAHNINYDAELERRRAERQMGS--GFHSSPV--GAGSVG 182
Pchry     RQHKEMMHSIREENEILKEILAANGIQYEADLERRRAERPPMM--AFQSSPVTVAGSSTT 163
Pmarn     QQQQETIQSLRTENNRLMGILKECGIPVQAQVEKETPEAIN---LAPGG-SFTSGPSSVA 184
Tstip     KQQQEMVQSLRTENNRLMGILKECGIPVQPQVEIETQETMN---AQLGATSYTVGTSSVA 173
                      **  *   *
```

B2 →

```
Trees    AHG-----------------SQGGFSPAG----TSHSTSMSMSPP--------------- 185
Tvire    APG-----------------SQSGFSPVG----TSHSTSMSMSP---------------- 180
Tatro    AHG-----------------SQGGFSPTG----MSQSTGMSMSP---------------- 175
Vdahl    THG-----------------SRSVFTPPL----TSKSTTASVSPSY-------------- 180
Moryz    GGG---PA----------SSHTSAFTPSP----LASDTANSQQYSR-------------- 176
Tterr    MAGSYIPA----------SSNTTALTPPP----LSASNG-SHGGGG-------------- 188
Ncras    MLG-----------------SESYLPGGP----SSQTTFPSPPSS--------------- 178
Ntetr    MLG-----------------SESYLPGGP----SSQTTFPSPPSST-------------- 179
Ndisc    MLG-----------------SESYLPGGP----SSQTTFPSPPSST-------------- 178
Foxys    TAP-------------------SVGSPMN----LPVAVNQGSTAG--------------- 186
Fvert    TAP-------------------SVGSPMN----LPVAVNQGSTTG--------------- 183
Fgram    TAP-------------------SVGSPMH----LPPAVNQGFVSGQ-------------- 185
Sscle    VPT-----------------SSSTFSPPP----LSHSSNSNPNSMN-------------- 183
Ptrit    GSA------------------STGYTSPP---TNGSASHDGMGGQG-------------- 217
Snodo    GSA------------------STGYTSPPSFQHRGSISHDSMGQPP-------------- 232
Chete    GSA------------------STGYTSPP---TRGSASHDGMSSQP-------------- 181
Mfiji    ASE------------------STGFSPPP----PTVPGQMPMPQG--------------- 164
Mgram    ASD------------------SAGFSPTP----DPIPG---MHQGQ-------------- 187
Abenh    PQIAPDL------------GSMNYLGTPETSIS----GRSPGTTT--------------- 158
Tverr    PQIAPDI------------GSMNYLGTPETSIS----GRSPGTTT--------------- 183
Trubr    SQTAPDI------------GNMNYLGTPEASIS----GRSPGTTT--------------- 203
Ttons    SQIAPDI------------GNMNYLGTPETSIS----GRSPGTTT--------------- 203
Mgyps    SQTAPDM------------ASMNYLGTPDTSIS----GRSPGTTI--------------- 220
Mcani    IQTAPDM------------GSMNYLGTPETSISA---GRSPGTTT--------------- 191
Cimmi    SAGYP---------------SGTHYMTPDTTVSA---GRSPGAPG--------------- 209
Cposa    SAGYP---------------SGTHYMTPDTTVSA---GRSPGAPG--------------- 209
Pb-01    S--QSHS--------QGFTRGGDPPSATPSSIMS---AISPGTGS--------------- 191
Pb-03    S--QSHSHVPSHSHSQGFTGGGNPPTSTPSTIMS---AISPGTGS--------------- 193
Aderm    APSQAPSQAHSHSLSQGFLGDGTYPTATP-TTVS---TVSPGTGS--------------- 196
Afumi    SQAP--AA--------LAASDGNTYTTPPTTVSNVSTDVSPLANG------------MER 207
Nfisc    SQAP--AA--------LAASNGNTYTTPPTTVSNVSSDLSPLAIG------------MER 177
Aclav    SQG---TG--------APTSNSHTHATPPTTISTLSSDVSPLANG------------MER 189
Aflav    SQPTAVAQ--------SVPSTQHAYTTPPTTISAPSSSLSPIVNG------------IEH 217
Aoryz    SQPTAVAQ--------SVPSTQHAYTTPPTTISAPSSSLSPIVNG------------IEH 217
Anige    SQPTGVAP--------SNPSTNNMYTTPPTTVSA---SLSPITTG------------IEQ 211
Aterr    SQPNGIA-----------PSASNVYTTPPTTVSASS-GVSPSVNGSTNGVDVSPTPHVSN 224
Anidu    SQTG------------IASLTAHTYSTPPTTVSS---GMSPKFNG------------VEK 215
Pchry    SQT---AP--------IAHSASNHDTTPATTISS---GMSPGANG------------MDH 197
Pmarn    SQSAG------------FQSQPAFLTTPPSTFSS------PHSAG-----------DGDE 215
Tstip    SQSAG------------FQSQPGFLTTPPSTFSS------PHSAGG----------DGDD 205
```

C236

← B3

```
Trees    -------------------DNRQLSPAA------------------NGADLEQA-GIDFV 207
Tvire    -------------------DNRQQSPAG------------------SGADLEQA-GIDFV 202
Tatro    -------------------DNRQPSPAG------------------NGVDHEQA-GIDFV 197
Vdahl    -----APNSGSSHGNTPSGQQQQQHPQHGTSQHLPGHYGLPCDDGVSGSRIRQLRGTARP 235
Moryz    -----TPTGHGSLSPHPGGHHQHTNAQHHPRHGA-------QPLNNPGVDYEQA-GIDFV 223
Tterr    -----GMSPHSAAYSHGGHHQHHPSVQGNPSAGAGV---GSHPNQNLNLDYEQA-GIDFV 239
Ncras    ------TTTSPAYRPPLGGPRDFNTVLG---DGAG----VMVSKRDPDLDYDQA-GIDFV 224
Ntetr    ------TTTSPVYRPSLGGPRDFNTVLG---DGAG----GLVSKRDPDLDYDQA-GIDFV 225
Ndisc    -----TTTTSPFYRPPLGGPSDFNTILG---DGAG----VLAPKRNPDLDYDQA-GIDFV 225
Foxys    ------------------QPHGMPQPLYR-----------------GNPELEQA-GIDFV 210
Fvert    ------------------QPHGMPQPLYR-----------------GNPGLEQA-GIDFV 207
Fgram    ------------------QQHGVPRPLYH-----------------GNPELEQA-GIDFV 209
Sscle    -----YDTESPQIGGANGVYNGNDRSMAQ--------------QQVRGVDYDQA-GIDFV 223
Ptrit    -----MTLQQQ-------AQQPNHHQH-------------------NGLDYDQI-GIDFV 245
Snodo    -----MALQQQ-------QVHQSGPQQ-------------------NGLDYDQI-GIDFV 260
Chete    -----LTLQHQ-------AQQQHHHQP------------------QGLDYDQI-GIDFV 210
Mfiji    -----IQNMQA-------GMVRNMAQMPS-----------------NRLDYDSI-GIDFV 194
Mgram    -----MAPPQS-------MPMRNMAQLPN-----------------NRLDYDQI-GIDFV 217
Abenh    SEMAQLPATGSNNPYFESAIPVAATGNALSSSDTG-GGSALVSSTPGVFDVDPQLGIDFV 217
Tverr    SEMAPLPATGSNNPYFESAIPVAATGNALSSSDTG-GGSALVSSTPGVFDVDPQLGIDFV 242
Trubr    SEMAQLPAVGSNNPYFESAIPVPATGNALSSSDIG-GGSALVSSTPGVFDVDPQLGIDFV 262
Ttons    SEMAQLPAAGSNNPYFESAIPVPATGNTLSSSDIG-GGSALVSSTPGVFDVDPQLGIDFV 262
Mgyps    SEMAQQPTAGSNNPYFESATPLPGTGYNTSSNSIG-GGTALVSSTPGVFDVDPQLGIDFV 279
Mcani    SETVQQPTAGSSNPYFESAAPQPGVSYQLSSNGFG-GESALVSSTPGVFDVDPQLGIDFV 250
Cimmi    SDFADAQPTSSPYSYQGPKTQKSCQPAISSEISIPSGDHSTVADVPGIFDRDPQLGIDFI 269
Cposa    SDFADAQPTSSPYSYQGPKTQKSCQPAISSEISIPSGDNSTVADIPGIFDRDPQLGIDFI 269
Pb-01    DSTETQPGGNMFPLYSESQGHQKEQSGVLAESSSWGGDGTAVDDVPGIFEKDPQLGVDFI 251
Pb-03    DSTETQPGGNMFPLYSESQGHQKEQSGVLAESSSWGGDGTAVDDVPGIFEKDPQLGVDFI 253
Aderm    DYTDPQGSGNIFTSYSGSQGIQSEQPGVLGESSAWGGDSSVVDDLPGIFEKEPELGVEFV 256
Afumi    VEISPTHEITPPPPIAMT-ASSCEIPADLDL-SAIARNQEPVQAVGGVFEADPQLQIDFI 265
Nfisc    VEISPTHEITPPPPIAMMTASSCEIPANLDL-SAIARNQEPVQAVGGIFEVDPQLQIDFI 236
Aclav    VEISPSQEITPPP--QSHHAGSCDIPANLDL-SAIARNIEPIQALGGVFETDPQLQVDFI 246
Aflav    IDVSPTQELSPQH--QNYSAAPCDALATLDR-IAPA-SRQPNQPPG-IFENDPQLQIDFI 272
Aoryz    IDVSPTQELSPQH--QNYSAAPCDALATLDR-IAPA-SRQPNQPPG-IFENDPQLQIDFI 272
Anige    IDVS-SSEMSPPQ--GPYQAAPCDALATLDQ-IAPV-SRAPRQPSG-IFEELPQLQIDFI 265
Aterr    IDISPTQEISPPQ--VAYNHAPCDALAALDR-IAPS-NRKPNQPPG-VFEEDPQLQIDFI 279
Anidu    FDFSSVPDVGPNN--QGIATVPCDALAAID-------RRPPIQGAG-IFEDNPQLQIDFI 265
Pchry    SDMSPPIGYPSHQ--QVYHANAGEHSMRMDPSCAPVDTMQPMPAKGGVFESDPQLQVDFI 255
Pmarn    RSGSRAGPGGG-MPIIGTTFQTGMGLSELDFSGSSEKERELLPSIPGIFDEDPQLGVDFI 274
Tstip    RAGSRSGTGGGAMPMIGTSFQTGMNLGDLDYSASSEKD-QAIPAVPGIFDEDPQLGIDFI 264
```

```
                   ------------------------  B3  ---------------------------->< B4
                       C272    C280                                 C303
Trees    LTLERPCMAHMPFLVDRASDADG---------------APCGHALMASCPPTPFQDLTP  251
Tvire    LTLEKPCMAHMPFLVDRASDADG---------------APCGHALMASCPPAPFQDLTQ  246
Tatro    LTLEKPCMAHMPFLVDRASDADG---------------APCGHALMASCPPAPFQELTP  241
Vdahl    MTLEKPCMNHMPWLLERSSEMGG---------------EPCGHALMASCPPEPFPELTP  279
Moryz    LAYENPHE------KQRGTETGG---------------EACGHALMASCPPEPFSELTP  261
Tterr    LTLEKPCMNHLPWMLERNSETGGR--------------EPCGHALMASCPPQPFSQLSS  284
Ncras    LTLERPCMEHMTWLLERGTGTEDRA-----------QREPCGHALMASCPPEPFSELSP  272
Ntetr    LTLERPCMEHMTWLLERGTGTEDRA-----------QREPCGHALMASCPPEPFSELSP  273
Ndisc    LTLERPCMEHMTWLLERGVGTEDRA-----------QREPCGHALMASCPPEPFSELSP  273
Foxys    LTLERPCMTHIQFMVERASEPEG---------------EPCGHALMASCPPDPAPETRP  254
Fvert    LTLERPCMTHIQFMVERASEPEG---------------EPCGHALMASCPPDPAPETRP  251
Fgram    LTLERPCMTHIQFMVERASEPEG---------------EPCGHALMASCPPDPSPESRP  253
Sscle    LTLERPCMDHMQFLVERSSDVDG---------------EFFSGHALMATCPPESDMDMHP  268
Ptrit    LTLERPCMDHMQFLMVRAHEAD---------------DNISGHALMATAPPDAHITNCP  289
Snodo    LTLERPCMDHMQFLMVRAHDAE---------------ETISGHALMATAPPDSHIAQCP  304
Chete    LTLERPCMDHMQFLMVRAHDAD---------------ENISGHALMATAPPDAHITNCP  254
Mfiji    LTLERPCMDHMQFLMVRSYNPDGKEFNHPMENPDDTEHDHMSGHALMATAAPYSHIMHKP  254
Mgram    LTLERPCMDHMQYLLVRSHNPTGQPHHHPMENADDALHEHMSGHALMASGPPLSHIIDRP  277
Abenh    LHLEKPCNWHLEFICRR--AHDDE-----------NQEAVSGHSLMASCPTSSVIANTE  263
Tverr    LQLEKPCNWHLEFICRR--AHDDE-----------NQEAVSGHSLMATCPTSSVIANTE  288
Trubr    LHLEKPCNWHLEFLCRR--AHDDE-----------NQEAVSGHSLMATCPTSSVIANTK  308
Ttons    LHLEKPCNWHLEFLCRR--AHDDE-----------NQEAVTGHSLMATCPTSSVIANTE  308
Mgyps    LHLEKPCNWHMEFLCRR--AHDDE-----------NQEAVSGHTLMATCPTSSVIANTE  325
Mcani    LHLEKPCNWHMEFLCRR--AHDDE-----------NQEAVSGHMLMATCPTPSVIANTE  296
Cimmi    LTLEGQCRDHIERLCRR--AHDAE-----------DQDMVAGHILMATCPPPSVVASAS  315
Cposa    LTLEGQCRDHIERLCRR--AHDAE-----------DQDMVAGHILMATCPPPSVVASAS  315
Pb-01    LSLEQSCRTHMELLCRR--AEDDE-----------EHETISGHVLMASCPPPTQIVSAE  297
Pb-03    LSLEQSCRTHMELLCRR--AEDDE-----------EHETISGHVLMASCPPPTQIVSAE  299
Aderm    LSLEQSCRTHMEFLCRR--AEDDA-----------ETETISGHVLMASCPPPTQIVSTE  302
Afumi    LTLESPCREHTDYLCRRSITEADD-----------EDMPFSGHALMATCPPPSYIANTT  313
Nfisc    LTLESPCREHTDYLCRRSVTEADD-----------EDMPFSGHALMATCPPPSYIANTT  284
Aclav    LTLEGPCREHTDYLCRRSITEADD-----------EDMPFSGHALMATCPPPSYIANTT  294
Aflav    LTLESPCREHTDYLCRRSITEADD-----------EDMPFSGHALMASCPPPSYIANTT  320
Aoryz    LTLESPCREHTDYLCRRSITEADD-----------EDMPFSGHALMASCPPPSYIANTT  320
Anige    LTLESPCREHTDYLCRRSITEADD-----------EDMPFSGHALMATCPPPSYIANTS  313
Aterr    LTLESPCREHTDYLCRRSITEADD-----------EDMPFSGHALMATCPPPSYIENTT  327
Anidu    LALESPCRDHTDYLCRRSITEADD-----------EDMPFSGHALMATCPPPSYIANTT  313
Pchry    LTLEGPCREHTDYLCRRSVTEADD-----------EDMPFSGHALMATCPPPSYIAKTT  303
Pmarn    LKLEAPCRVHTEYLCREAHKDVE------------RDAFISGHALMASCPPPSHIENVP  321
Tstip    LQLESPCRDHTEYLCREASKDVE------------RDRFFSGHALMASCPPPNHIENVP  311
         :  *         .                        ** ***: ..
```

```
                  -------------------------  B4  --------------------------

Trees    DTPFGHTHTHHHGD-----------------------------GDGGGEEPLTQG----  277
Tvire    ETPFGNTHTHDHG---------------------------------GEFHPQSQG----  268
Tatro    DTPFGSKHTHDHG---------------------------------GEFEK--QG----  261
Vdahl    DIPFG--YAHVNG----------------------------------DLNS-GQR----  297
Moryz    DIPFGYSN------------------------------------VKNGELDSGQR----  280
Tterr    DIPFSHHQHQL--------------------------HQPQQQAQQQQRLDSQNNNNNT  317
Ncras    ESPFGHTNTIH-------------------------AHAHGHPPMPTSISGGGQR----  302
Ntetr    ESPFGHTNTIH-------------------------AHGHGHPPMPTSISGGGQR----  303
Ndisc    ESPFGHNNTIQNLAIGGNSNGDGGAITPIRQQHVHTHGHGHDYPPMPTSISGSGQR----  329
Foxys    GLPFGKTHIPLDG-------------------------------------DTNQK----  272
Fvert    GLPFGKTHIPLDG-------------------------------------DTNQK----  269
Fgram    GLPFGKIHIPVDG-------------------------------------EPSQK----  271
Sscle    HIPFGHCMPHNHN---------------------------HETDGTDGGPPKQK----  295
Ptrit    EEKYPHQMP--------------------------------------------------  298
Snodo    EEKYPHQMP--------------------------------------------------  313
Chete    EEKYPHQMP--------------------------------------------------  263
Mfiji    TEKYPHQMPE-------------------------------------------------  264
Mgram    AEPYPHQMPE-------------------------------------------------  287
Abenh    RGQTYETKT--------------------------------------------------  272
Tverr    RGQTYETKT--------------------------------------------------  297
Trubr    RGQTYETKT--------------------------------------------------  317
Ttons    RGQTYETKT--------------------------------------------------  317
Mgyps    RGQTYVTKT--------------------------------------------------  334
Mcani    RGQTYTTKT--------------------------------------------------  305
Cimmi    RGYEYPVKT--------------------------------------------------  324
Cposa    RGYEYPVKT--------------------------------------------------  324
Pb-01    PGQMYSTRT--------------------------------------------------  306
Pb-03    PGQMYSTRT--------------------------------------------------  308
Aderm    PGQMYPVRT--------------------------------------------------  311
Afumi    PEQAYPHKT--------------------------------------------------  322
Nfisc    PEQAYPHKT--------------------------------------------------  293
Aclav    AEQTYPHKT--------------------------------------------------  303
Aflav    HEQAYPHQT--------------------------------------------------  329
Aoryz    HEQAYPHQT--------------------------------------------------  329
Anige    NEQTYPHKT--------------------------------------------------  322
Aterr    DKQVYPHKT--------------------------------------------------  336
Anidu    SEQTYPHKT--------------------------------------------------  322
Pchry    PEQPYPHKA--------------------------------------------------  312
Pmarn    EGNLYPHQT--------------------------------------------------  330
Tstip    EGNLYPHQT--------------------------------------------------  320
```

B4

(C354)

```
Trees    -------------------TWELTKADLTTLLDLSQKLN-----LDGEITPVMAWGMVLS 313
Tvire    -------------------TWELTKADLSTLLDLSRKLN-----LDGEITPVMAWGMVLS 304
Tatro    -------------------TWELTKADLTTLLDLSKQLN-----LDGEITPVMAWRMVTS 297
Vdahl    -------------------TWELSKADLGTLLALSKRLN-----LDGEITPVMAWGMVMA 333
Moryz    -------------------TWELSKGDLNTLLDLSKRLN-----LDGEITPVMAWGMVLA 316
Tterr    TNDSNYNVNIDNDGSSTPGTWTLNKGDLATLLDLSRRLN-----LDGEITPVMAWGMVLA 372
Ncras    -------------------TWELSKADLATLLDLSKRLD-----LDGEITPVMAWGMILA 338
Ntetr    -------------------TWELSKADLATLLDLSKRLD-----LDGEITPVMAWGMVLA 339
Ndisc    -------------------TWELSKADLATLLDLSKRLD-----LDGEITPVMAWGMVLA 365
Foxys    -------------------TWEVPKADLATLLDLSKSID-----LDGEVTPIMSWGMLMS 308
Fvert    -------------------TWEVPKADLATLLDLSKSID-----LDGEVTPIMSWGMLMS 305
Fgram    -------------------TWEVPKADLATLLDLSKSID-----LDGEVTPIMSWGILMS 307
Sscle    -------------------TWDLSKSDLANLLDLSKRLD-----LNGEITPVMAWGMVLA 331
Ptrit    ---------------------DVKMPDLMKLLDLSNRLP-----LDGEITPIMAWAKIIQ 332
Snodo    ---------------------DITMPDLMKLLDLSNRLP-----LDGEITPIMAWAKIIQ 347
Chete    ---------------------DVNMPDLMKLLDLSNRLP-----LDGEITPIMAWAKILQ 297
Mfiji    ---------------------DLDNPTLVKLLDLSNRLP---LDHQGEITPVMAWKLIYL 300
Mgram    ---------------------EMSPTALGKLLDLSSRLPRNMYDREGEITPIMAWAMIFG 326
Abenh    --------------------YDLPPANLNTLLNLSKQLVT-----DDEITPIMALQLLRN 307
Tverr    --------------------YDLPPANLNTLLNLSKQLVT-----DDEITPIMALQLLRN 332
Trubr    --------------------YDLPPANLNTLLNLSKQLVT-----DDEITPIMALQLLRN 352
Ttons    --------------------YDLPPANLNMLLNLSKQLVT-----DDEITPIMALQLLRN 352
Mgyps    --------------------YDLPPANLNTLLNLSRQLVT-----DDEITPIMALQLLRN 369
Mcani    --------------------YDLPPVNLNTLLNLSKQLVT-----DDEITPIMALQLLRS 340
Cimmi    --------------------YDLPPANLNTLLNLSRQLVT-----DNEITPIMALQSLKN 359
Cposa    --------------------YDLPPANLNTLLNLSRQLVT-----DNEITPIMALQSLKN 359
Pb-01    --------------------YDLPHANLTALLNLSRQLVT-----GGQITPIMALQYLKS 341
Pb-03    --------------------YDLPHANLTALLNLSRQLVT-----GGQITPIMALQYLKS 343
Aderm    --------------------YDLPHSNLTRLLNLSRQLVT-----DGQITPIMALQYLKS 346
Afumi    --------------------YDLPHANLTTLLNLSRQLVT-----EGQITPIMALQCLKN 357
Nfisc    --------------------YDLPHANLTTLLNLSRQLVT-----EGQITPIMALQCLKN 328
Aclav    --------------------YDLPHANLTTLLNLSRQLVT-----EGQITPIMALQCLKN 338
Aflav    --------------------YDLPHANLTTLLNLSRQLVT-----DGQITPIMALQCLKN 364
Aoryz    --------------------YDLPHANLTTLLNLSRQLVT-----DGQITPIMALQCLKN 364
Anige    --------------------YDLPHANLSTLLNLSRQLVT-----DGQITPIMALQCLKN 357
Aterr    --------------------YDLPLANLSTLLNLSRQLVT-----DGQITPIMALQHLKN 371
Anidu    --------------------YDLPHANLTTLLNLSRQLVT-----EGQVTPIMALQALKN 357
Pchry    --------------------PDLPHANLSTLLNLSRQLVT-----EGQITPIMALQCLKN 347
Pmarn    --------------------YDLPLPNLGKLLNLSKQLIT-----EGQVTPIMILQSLKN 365
Tstip    --------------------YELPLPNLEKLLNLSKQLIT-----DGQVTPIMILQSLKN 355
                                :    *  ** **  :        .::**:*    :
```

B4

C382 C397

```
Trees    HARFAELAPCDFEKISGELLRKVRCYGFGAVLEEFELRDAIENIFSGRPEA------MVF 367
Tvire    HSRFAELESGDFEKLSEELLRKIRCYGFGAVMEEFELRDALENIFSGRPEA------MVF 358
Tatro    HYRFNELAPGDLEKLSEELLRKIRCYGFGAVMEEFELRDALENIFSGRPEA------MVF 351
Vdahl    HSRFYEMKAEDFAKLTEELSTKIRCYGFGAVLEEFEIRDALENAMGAYP----------- 382
Moryz    HPRLGEMTPKDFKKLTEELCGKVRCYGFGAVMEEFEARDAIENVFSTKPDSFHQSYSTEY 376
Tterr    HPRLGELRAEDFARLAEELLGKVRCYGFGAVLEEFEVRDALENIFSTKPD-----FGTGY 427
Ncras    HPRLAELQLEDFARLTEELGSKVRCYGFGAVMEEFEVRDALENLFSTKPDG---RVFEDF 395
Ntetr    HPRLAELQLEDFARLTEELGSKVRCYGFGAVMEEFEVRDALENLFSTKPDG---RVFEDF 396
Ndisc    HPRLAELQLEDFARLTEELGRKVRCYGFGAVMEEFEVRDALENLFSTKPDG---RVFEDF 422
Foxys    HPRANELEAVDFRKLSEELVRKVRCYGFGAVMEEFEVRDAIDNVFSGKDTMMGE------ 362
Fvert    HPRANELEVVDFRKLSEELVRKVRCYGFGAVMEEFEVRDAIDSVFSGKDSMMGE------ 359
Fgram    HPRANELEAADFRKLSEDLVRKVRCYGFGAVMEEFEVRDAIDNVFSGKDGMMYE------ 361
Sscle    HPRFLELKEEDFKCMSAELLPKVRCYGFGAALEEFEVRDALEALFGAELVHMSSI----- 386
Ptrit    NENFRTLSKEDIELMKGELLAKVRCYGFGAVLEEFEVSDALMTVLAGKFSNG-ATPLA-- 389
Snodo    DANFQDLTKDEIEVIKTELLAKVRCYGFGAVLEEFEVSDALMTVLAGKYSNGNPTPTA-- 405
Chete    HDKLRDLSKEDFELIKGELLAKVRCYGFGAVLEEFELRDALTTVLAGKFSNG-SPPIG-- 354
Mfiji    NERVRDLSKFDFDRMKTDLATKVRCYGFGAVLEEFEVRDALDGILAEKDGQMMPALDQKM 360
Mgram    HERVNELVEEDVERVKKGLEGKVRCYGFGAVVEEFEVRDAIAAVFNDKDSVMRG------ 380
Abenh    HDAYPYLTREDIAHIMSDLAAKVRCYGFGAVLENFEVMDSLNSVLTSKMDYAIAS-CEGS 366
Tverr    HDAYPYLTREDIAHIMSDLAAKVRCYGFGAVLENFEVMDSLNSVLTSKMDYAIAS-CDGS 391
Trubr    HDAYPYLTREDVAHIMSDLAAKVRCYGFGAVLENFEVMDSLNSVLTSKMDYAIAS-YDGS 411
Ttons    HDAYPYLTREDVAHIMSDLAAKVRCYGFGAVLENFEVMDSLNSVLTSKMDHAIAS-YDGS 411
Mgyps    HEAYPYLTREDVAHMMSDLAAKVRCYGFGAVLENFEVMDSLNSVLTSKMDYAIAS-YDGS 428
Mcani    HEAYPYLTREDVTNMMDDLAAKVRCYGFGAVLENFEVMDSLNSVLTSKMDYAFAS-PDGS 399
Cimmi    HEMYHLLTKDDIRRVMDDLVAKVRCYGFGAVLEEFEFRDSLNSVLGSKMEPAIMQ-AEG- 417
Cposa    HEMYHLLTKDDIRRVMDDLVAKVRCYGFGAVLEEFEFRDSLNSVLGSKMEPAIMQ-AEG- 417
Pb-01    HDTYPSLTREDVRHMMDDLNGKIRCYGFGAVMEDFEFMDSFSSVLARKMEPPMDTGPDDL 401
Pb-03    HDTYPSLTREDVRHMMDDLNGKIRCYGFGAVMEDFEFMDSFSSVLARKMEPLMDTGPEDL 403
Aderm    HDMYPSLTREDVENMMDDLNGKIRCYGFGAVLEDFEFMDSFSSVLASKLKSLIDTAPDDP 406
Afumi    HELYRTLTKDDVKIIIETLNTKVRCYGFGAVVEDFELMDCLSSVLGSKVDTS-------- 409
Nfisc    HELYRTLTKDDVKVIIETLNTKVRCYGFGAVVEDFELMDCLSSVLGSKVDTR-------- 380
Aclav    HDMYRNLTKDDVKIIIDTLNTKVRCYGFGAVVEDFELMDCLSSVLGSRVGPG-------- 390
Aflav    HEMYRSLTRDDVKIIIETLNTKVRCYGFGAVVEDFELMDCLSSVVGSRVDMG-------- 416
Aoryz    HEMYRSLTRDDVKIIIETLNTKVRCYGFGAVVEDFELMDCLSSVVGSRVDMG-------- 416
Anige    HELYRSLTKEDVKVIIETLNTKVRCYGFGAVVEDFELMDCLSSVLGSKVEGA-------- 409
Aterr    HELYRTLTKDDVKIIIETLNTKVRCYGFGAVVEDFELMDCLSSVLGGKVDVG-------- 423
Anidu    HEMYTKLTRDDVKLIMDTLENKVRCYGFGAVLEDFELMDCFSSVLGSKVEGSGPV----- 412
Pchry    HELYPTLRRDDIKIIIDTLNTKVRCYGFGAVVEDFELIDCLSSVLGTKVDLG-------- 399
Pmarn    HEQYRSLTKEDVKTIVETLTAKVRCYGFGAVIEDFELRDCLQNVLGAKLYHGRR------ 419
Tstip    HDQYRHLTKDDVKAIVDTLTAKIRCYGFGAVIEDFELRDCLQNVLGTKLYHGRS------ 409
         .     :   :.  :   *  *:*******.:*:**  *.:   .
```

```
Trees    ------------------
Tvire    ------------------
Tatro    ------------------
Vdahl    ------------------
Moryz    GGGR-------------- 380
Tterr    ------------------
Ncras    ------------------
Ntetr    ------------------
Ndisc    ------------------
Foxys    ------------------
Fvert    ------------------
Fgram    ------------------
Sscle    ------------------
Ptrit    ------------------
Snodo    ------------------
Chete    ------------------
Mfiji    AAQQISVH---------- 368
Mgram    ------------------
Abenh    SSLISPPIQSADVLDMYS 384
Tverr    SSLISPPIQSADVLDMYS 409
Trubr    SSLISPPIQSADVLDMYS 429
Ttons    SSLISPPIQSADVLDMYS 429
Mgyps    SSLISPPIQSSEVLDMYS 446
Mcani    SSLISPPIQSAEVLDMYS 417
Cimmi    -YLPFRPAHVAEDYTMYS 434
Cposa    -YLAFRPAHVAEDYTMYS 434
Pb-01    KPLPPIL----------- 408
Pb-03    KPLPPIL----------- 410
Aderm    ASLPATPRQRLSDDLMYS 424
Afumi    -------FSCAGDDTMYS 420
Nfisc    -------FSCAGDDTMYS 391
Aclav    -------MSLAADD---- 397
Aflav    -------FSRAGDDTLYS 427
Aoryz    -------FSRAGDDTLYS 427
Anige    -------LSHPGDEMMYS 420
Aterr    -------FSRSEDHMLYS 434
Anidu    ----GVAFSRHADETMYS 426
Pchry    -------MSGTADDSMYG 410
Pmarn    ------PPSRIGDDALYR 431
Tstip    ------PPSKVGDDALYR 421
```
