# Supplementary material for: Elucidation of Functional Markers from Aspergillus nidulans Developmental Regulator FlbB and Their Phylogenetic Distribution
Source: PLoS One. 2011 Mar 10;6(3):e17505. doi: 10.1371/journal.pone.0017505 (PMC3053368; doi:10.1371/journal.pone.0017505)
Supplement: Figure S2 — Alignment of the 40 Pezizomycotina FlbE orthologs used in this study. Anidu FlbE is in bold. Conserved motifs E1, E2, E3, E4 and E5 are labeled and highlighted in green or yellow. The acidic region is highlighted in purple. Residues flanking intron locations are in bold italic. (PDF) [file pone.0017505.s002.pdf]

Additional Figure 2 (page 1 of 10)

```
         <------------------- E1 ------------------->  <-- E2 -->
Ncras    MPTYLCHGFRWQRRS------IRVYTVVQDLDDVSPEWLIRPTSSRCLLQSFYNLFEFLP 54
Ntetr    MPTYLCHGFRWQRRS------IRVYTVVQDLDDVSPEWLIRPTSSRCLLQSFYNLFEFLP 54
Ndisc    MPTYLCHGFRWQRRS------IRVYTVVQDLDDVSPEWLIPPNSSRCLLQSFYNLFEFLP 54
Foxys    MPTYLCHGFRWHRRD------IRIFVILNDLEDAAPNWVLAPASSYCILDQLHAKYDFLP 54
Fvert    MPTYLCHGFRWHRRD------IRIFVILNDLEDAAPNWVLAPASSYCILDQLHAKYDFLP 54
Fgram    MPTYLCHGFRWHRRD------IRIFVILNDLEDAAPNWILAPASSYCILDQLHAQFDFLP 54
Trees    MPTYLCHGFRWTRRL------IRIFVIIEDLDDAAPDWITGRNSSAAILERFGTKFDYLP 54
Tvire    MPTYLCHGFRWTRRL------IRIFVILEDLDDAAPDWITGRATSAVILERLGTKFDYLP 54
Tatro    MPTYLCHGFRWTRRL------IRIFVILEDLEDAAPDWIVGRDTSAIILERMNTKFDYLP 54
Vdahl    MPTYLCHGFRWHRPS------IRVYVVVQNIDDAAPEWIIGPESSNAILESFYTNFDFLP 54
Moryz    MPTYLCHGFRWHRPS------IRVYVIVQDIGDAAPDFISGTRSATAILDSFYNLFDFLP 54
Tterr    MPTYLCHGFRWQRRS------IRVYVILQDLDDASPEWIIPAKSARCILQSFYDVFEFLP 54
Sscle    MPTYLVHGFRWHRTN------IRIHIILNDIEDASPEWLMAPRTSNALLNSFYTQFDFLP 54
Nfisc    MPVYMVHGFRWPRAG---FTGIRVYIVLHNLEDATAEYVQQPATSRLLTECFKKTDPDIV 57
Afumi    MPVYMVHGFRWPRAG---FTGIRVYIVLHNLEDATAEYVQQPVTSRLLTECFKKTDPDIV 57
Aclav    MPVYMVHGFRWPRIG---FTGIRVYIVLHNLEDATAEYVQQPETNRLLTECFKKTDPDIV 57
Anige    MPVYMLYGFRWPRAG---FTGIRVYIVLHNLEDATAEYLQQPLTSELVLESLKKTEPGIV 57
Anidu    MPVYMLYGFRWPRAG---FTGIRVYIVLHNLEDATAEYIQRPITNKSLLDSFRKTEPDIM 57
Aoryz    MPVYMLYGFKWPRAG---FTGIRVYIVLHNLEDATAEYLQAPVTTQLLLESFAKTEPGIV 57
Aflav    MPVYMLYGFKWPRAG---FTGIRVYIVLHNLEDATAEYLQAPVTTQLLLESFAKTEPGIV 57
Aterr    MPVYMLYGFRWPRAG---FTGIRVYIVLHNLEDATAEYLQQPVTHQLMLDSLKKTEPGIV 57
Pchry    MPVYMLHGFRWPRAG---FTGIRVYIVLHNLEEAAAEYIQQPLTTELIAESFHKTQADLV 57
Pmarn    MPVYKIHGFPWPRGG---IPSIRVFIVLENIDDASAEYIQNKRTSNLILNAIKKNHPAAE 57
Tstip    MPVYKIHGFPWPRGG---IPSVRVFIVLENLDDAAAEYIQQKKTSDLILGAIRKNHPDAV 57
Cimmi    MVVYVVHGFRWPRVGTLNAPGICAHIVSYNLLDAAAEYLQEPATCRAILQSFRRIDPNIP 60
Cposa    MVVYVLHGFRWPRVGTLNAPGICAHIVSYNLLDAAAEYLQEPATCRAILQSFRRIDPNIP 60
Ttons    MVVYIVYGFRWSRVGTLTAPGIRPHIVINDLLDAASDYIQEPRTAKEVVQSFERIDANIP 60
Trubr    MVVYIVYGFRWSRVGTLTAPGIRPHIVINDLLDAASDYIQEPRTAKEVIQSFGRIDANIP 60
Tverr    MVVYIVYGFRWSRVGTLTAPGIRPHIVINDLLDAASDYIQEPRTAKEVIQSFERIDANIP 60
Abenh    MVVYIVYGFRWSRVGTLTAPGIRPHIVINDLLDAASDYIQEPRTAKEVIQSFERIDANIP 60
Mgyps    MVVYIVYGFRWSRVGTLTAPGIRPHIVINDLLDAASDYIQEPRTAKEVIQSFERIDANIP 60
Mcani    MVVYIVYGFRWSRVGTLAAPGIRPHIVINDLLDAASDYIQEPRTAGEVLKSFERIDPSIP 60
Pb-01    MVTYILYGFRWNRIASPSAYGIRAYIAIANIADAAAEYLQHPITNAVILDSFNHIDPNIL 60
Pb-03    MVTYILYGFRWNRIASPSAYGIRAYIAIANIVDAAAEYLQHPITNAVILDSFKHIDPNIL 60
Aderm    MVTYILYGFRWHRAPNPLAPGIRAYIALYNLLDAAAEYLQHPTTTAAVLNSFKLLDPDIL 60
Mfiji    MPVYLLHGFRWPRPL------VRIHIILKNLDDAAAEWLVAPGTTKALLDNFQELFPECM 54
Mgram    MPVYLLHGFRWPRPL------IRIHIILQNLDDAAAEWLVAPGTTETMLENFHETFPDTM 54
Ptrit    MPTWLVHGFRWPRAQ------IRIHVILQNLDDAAPEWLMAPATTACMTENFKKQWPEQM 54
Chete    MPTWLVHGFRWPRAQ------IRIHIILQNLDDAAPEWLMAPATVACMTKNFKDQWPETM 54
Snodo    MPTWIVHGFRWPRAQ------IRIHIILQNLDDAAPEWLMAPATVACMTANFKQQWPEEM 54
         * .:  :** * *        :  .    :: :.:.:::    :   :   :

         <--------------------------- E3 --------------------------->
Ncras    YCSPHEPETWDSNED--------EDEDEDEDEGDKSGEENGEE---EAVVVPGSRGSRGE 103
Ntetr    YCSPHEPETWDSNED--------EDEDED--EGDESGQENGEDNDGQGAVVPDNRGERGN 104
Ndisc    YCSPYEPGTWDSQEDEVETETETENENEDEDEGEGEGNESGKE-IVEGVVAGSRRRRCGE 113
Foxys    ELSPPT-------------------------TPTQKSIS--------------------- 68
Fvert    ELSPPT-------------------------TPTQKSVS--------------------- 68
Fgram    ELTPPT-------------------------TPTQASFSN-------------------- 69
Trees    QRLPQQ-------------------------QAETPSEQESVP----------------- 72
Tvire    QRVPPQ-------------------------QPEQKLEN--------------------- 68
Tatro    QRLPPQPDEE---------------------QSEQSKQQSEPQ----------------- 76
Vdahl    EARPASGD-----------------------RQQQQRKKAAAG----------------- 74
Moryz    --PPK------------------------------------------------------- 57
Tterr    YCSPKRGRHAPARDVDGGDDTHRALGYANDAPRDRSRSRGRGR----------------- 97
Sscle    PSYPGP-----------------------------IDLSAESA----------------- 68
Nfisc    ARLPD------------------------------------------------------- 62
Afumi    ARLPD------------------------------------------------------- 62
Aclav    AHLPD------------------------------------------------------- 62
Anige    SRLPN------------------------------------------------------- 62
Anidu    SNLPE------------------------------------------------------- 62
Aoryz    SRLPD------------------------------------------------------- 62
Aflav    SRLPD------------------------------------------------------- 62
Aterr    SRLPD------------------------------------------------------- 62
Pchry    NRLPE------------------------------------------------------- 62
Pmarn    KHLPN------------------------------------------------------- 62
Tstip    KHLPN------------------------------------------------------- 62
Cimmi    YHLPD------------------------------------------------------- 65
Cposa    YHLPD------------------------------------------------------- 65
Ttons    SHLPN------------------------------------------------------- 65
Trubr    SHLPN------------------------------------------------------- 65
Tverr    SHLPN------------------------------------------------------- 65
Abenh    SHLPN------------------------------------------------------- 65
Mgyps    SHIPD------------------------------------------------------- 65
Mcani    AHLPD------------------------------------------------------- 65
Pb-01    THIPD------------------------------------------------------- 65
Pb-03    THLPD------------------------------------------------------- 65
Aderm    THLPN------------------------------------------------------- 65
Mfiji    THLPD------------------------------------------------------- 59
Mgram    KHLPN------------------------------------------------------- 59
Ptrit    AQLPD------------------------------------------------------- 59
Chete    AQLPT------------------------------------------------------- 59
Snodo    KKLPE------------------------------------------------------- 59
            *
```

```
                               E3
Ncras    KGSEEQRTQWPKRKESLGVLRGHGSEGTTGKGMGTMGKEMGKGMGKEKREITA-IPVGS- 161
Ntetr    ----EQRTQWPKRKESLGVLRGHGSEGTTGKGMGTMGKEMGKGMGKEKREITA-IPVGS- 158
Ndisc    SGNEEPMRQWPKRKESLGVLRGHGSEEGKGKVMQMGKGTMGKGT-TGKGEITANIHVGSS 172
Foxys    ------------------------------------------------------------
Fvert    ------------------------------------------------------------
Fgram    ------------------------------------------------------------
Trees    ------------------------------------------------------------
Tvire    ------------------------------------------------------------
Tatro    ------------------------------------------------------------
Vdahl    ------------------------------------------------------------
Moryz    ------------------------------------------------------------
Tterr    ------------------------------------------------------------
Sscle    ------------------------------------------------------------
Nfisc    ------------------------------------------------------------
Afumi    ------------------------------------------------------------
Aclav    ------------------------------------------------------------
Anige    ------------------------------------------------------------
Anidu    ------------------------------------------------------------
Aoryz    ------------------------------------------------------------
Aflav    ------------------------------------------------------------
Aterr    ------------------------------------------------------------
Pchry    ------------------------------------------------------------
Pmarn    ------------------------------------------------------------
Tstip    ------------------------------------------------------------
Cimmi    ------------------------------------------------------------
Cposa    ------------------------------------------------------------
Ttons    ------------------------------------------------------------
Trubr    ------------------------------------------------------------
Tverr    ------------------------------------------------------------
Abenh    ------------------------------------------------------------
Mgyps    ------------------------------------------------------------
Mcani    ------------------------------------------------------------
Pb-01    ------------------------------------------------------------
Pb-03    ------------------------------------------------------------
Aderm    ------------------------------------------------------------
Mfiji    ------------------------------------------------------------
Mgram    ------------------------------------------------------------
Ptrit    ------------------------------------------------------------
Chete    ------------------------------------------------------------
Snodo    ------------------------------------------------------------
```

```
                               E3
Ncras    -----NNRSSISRTRTISRSPSLRLPLQGVRTP--SRTRLQTTAAAAASAAT-------- 206
Ntetr    -----NNRSSISRTRTISRSPSLRLPLQGARTP--SRTRLQATAASAATAPT-------- 203
Ndisc    NNNNNNNNNNNNNRRTTSRSPSLRLPLQGARTPSTSRTRQTATVAAAAAAITPTTPTTPA 232
Foxys    ------AKNANIKKQFDHVDDDHALPTSRVS----------------------------- 93
Fvert    ------AKNANIKKQFDHVDDDHALPTSRVS----------------------------- 93
Fgram    ------AKNIKPKPKPDHVDDDHDLPKSRVP----------------------------- 94
Trees    ---QQQKQATQDEKIAAHQDDDFSVPPSRVP----------------------------- 100
Tvire    ----QQGQAKQEEKKIAHQDDNLTVPPSRVP----------------------------- 95
Tatro    ---EEQPPPEQEEKKFVHQDDDLTVPPSRVP----------------------------- 104
Vdahl    ---TGASKRRNGSQAHNDAADPFAKPPPPVP----------------------------- 102
Moryz    -----------------TRDDQQKQPP--------------------------------- 67
Tterr    ---GRSQSQSTAQSRSLSRAKRQQQPPPSLPTS--------------------------- 127
Sscle    ---PRESDYLPPSSPSRTLTKKSNQSPASLK----------------------------- 96
Nfisc    ------------------------------------------------------------
Afumi    ------------------------------------------------------------
Aclav    ------------------------------------------------------------
Anige    ------------------------------------------------------------
Anidu    ------------------------------------------------------------
Aoryz    ------------------------------------------------------------
Aflav    ------------------------------------------------------------
Aterr    ------------------------------------------------------------
Pchry    ------------------------------------------------------------
Pmarn    ------------------------------------------------------------
Tstip    ------------------------------------------------------------
Cimmi    ------------------------------------------------------------
Cposa    ------------------------------------------------------------
Ttons    ------------------------------------------------------------
Trubr    ------------------------------------------------------------
Tverr    ------------------------------------------------------------
Abenh    ------------------------------------------------------------
Mgyps    ------------------------------------------------------------
Mcani    ------------------------------------------------------------
Pb-01    ------------------------------------------------------------
Pb-03    ------------------------------------------------------------
Aderm    ------------------------------------------------------------
Mfiji    ------------------------------------------------------------
Mgram    ------------------------------------------------------------
Ptrit    ------------------------------------------------------------
Chete    ------------------------------------------------------------
Snodo    ------------------------------------------------------------
```

```
                 ------------------------------ E3 -----------------------------
Ncras    TPTTPTAPTAAIPAPASTPALRTASTSGVSSAGKG-RKGKTTGNSSSSR----RSESHSP 261
Ntetr    TPTAPTTATTTTSAPASTPASRTTSALGVSSAGKG-RKGKTTGNSSSSRSSSRRSASHSP 262
Ndisc    TPATTTTTTTTTTASAPASSTRTTSTLGVPSGARGGRKGKMTGSSSSSNSS--RSASHSP 290
Foxys    ------------------------------------------------------------
Fvert    ------------------------------------------------------------
Fgram    ------------------------------------------------------------
Trees    ------------------------------------------------------------
Tvire    ------------------------------------------------------------
Tatro    ------------------------------------------------------------
Vdahl    ------------------------------------------------------------
Moryz    ------------------------------------------------------------
Tterr    --------------------------------------------------------DDAS 131
Sscle    ------------------------------------------------------------
Nfisc    ------------------------------------------------------------
Afumi    ------------------------------------------------------------
Aclav    ------------------------------------------------------------
Anige    ------------------------------------------------------------
Anidu    ------------------------------------------------------------
Aoryz    ------------------------------------------------------------
Aflav    ------------------------------------------------------------
Aterr    ------------------------------------------------------------
Pchry    ------------------------------------------------------------
Pmarn    ------------------------------------------------------------
Tstip    ------------------------------------------------------------
Cimmi    ------------------------------------------------------------
Cposa    ------------------------------------------------------------
Ttons    ------------------------------------------------------------
Trubr    ------------------------------------------------------------
Tverr    ------------------------------------------------------------
Abenh    ------------------------------------------------------------
Mgyps    ------------------------------------------------------------
Mcani    ------------------------------------------------------------
Pb-01    ------------------------------------------------------------
Pb-03    ------------------------------------------------------------
Aderm    ------------------------------------------------------------
Mfiji    ------------------------------------------------------------
Mgram    ------------------------------------------------------------
Ptrit    ------------------------------------------------------------
Chete    ------------------------------------------------------------
Snodo    ------------------------------------------------------------


           ---------- E3 --------->      <--- E4 --->
Ncras    -NRKKDELSAQRWSAVKVLEEYDPNN---LDEVSRPYAYVADHVVRVDGAADILAEVRRY 317
Ntetr    -NRKKDELSAQRWSAVKVLEEYDPNN---LDEVSRPYAYVADHVVRVDGAADILAEVRRY 318
Ndisc    PNHKKDELSAQRWSAVKVLEEYDPNN---LDEVSRPYAYVADHVVRVDGAADILAEVRRY 347
Foxys    --DAEDAVLMHSWSPVKLLEEFDVDE---MVTACRPYAYVADYVVRVDLSVDVAGQMAKY 148
Fvert    --DAEDAVLMHSWSPVKLLEEFDVDE---MVTACRPYAYVADYVVRVDLSVDVAGQMAKY 148
Fgram    --DAEDAVLMHSWSPVRLLEEFDVDE---MTMACRPYAYVADHVVRIDLSVDVAGEMAKY 149
Trees    --YSEDSVLVNQFSPVKLLEEYDPEE---TSISARPFAYVADHVIRIDLSANVLEEMAKY 155
Tvire    --DSEDSVLVNQSSPVKLLEEYDPEE---TSISARPFAYVADHVIRIDLSVNVLEEMARY 150
Tatro    --DSEDSVLVNQTSAVKLLEEHDPEE---TTISARPYAYVADHVIRIDLSADVLQEIANY 159
Vdahl    --PEEDDVLLNSWSAVKLLEEYDPTD---LTSVSRPYAFVADHVVRVDLSVSVVEEMAGY 157
Moryz    --AATDSASSQAWSAIRLLEEYDPSM---LGEAARPHAYVADHVVRVDLSASVVDEMARY 122
Tterr    SVVSEDDFSAQAWSAIKLLEEYDPRD---LTAVSRPYAYVADYAARIDLSCSIVDEIARY 188
Sscle    ---SPSPNDKPSLTLKKITSHRRNKN---SFESEALHSRSASGSTNPNSSGPQAKEMEKS 150
Nfisc    ---------------LQFIEQYDPEDTDSATAVSQPYAYVAAKVITLSEPGAK--APGLS 105
Afumi    ---------------LQFIEQYDPEDTDSATAVSQPYAYVAAKVITLSEPGAK--APGLS 105
Aclav    ---------------LRFIEQYDPEDTTSEMAVSQPYAYVAAKVITLSEPGAR--APALS 105
Anige    ---------------LRFIEQYDPED-TSDDAVSKPYAYVGGKVISIPEDVGP--TPETS 104
Anidu    ---------------LRFIEQYDPED-ESDEAVSKPYAYVAAKTISIPEAGSP--NAGSS 104
Aoryz    ---------------LRFIEQYDPDD-TSDEAVSKPYAYVAAKVITMPESG------ALS 100
Aflav    ---------------LRFIEQYDPDD-TSDEAVSKPYAYVAAKVITMPESG------ALS 100
Aterr    ---------------LRFIEQYDPDDTTSETAVSKPFAYVAGKVLTMPDMTSA--GMSLG 105
Pchry    ---------------LSFIEQYDPAD-ETSGTVSQDHAYVSTRVLEIPEDGSD--GGGGA 104
Pmarn    ---------------LQLIEQYDPLD-MTDAAVSQPHAFVADSVTILPDTARP--VRGLS 104
Tstip    ---------------LQLIEQYDPLD-MTDAAVSQPYAFVADNVTVLPDTARP--VQGLS 104
Cimmi    ---------------LRIIEQYDPDD-IGDTALRQPYAFVAAKVITMPDEGRP--GQMLS 107
Cposa    ---------------LRIIEQYDPDD-IGDTALRQPYAFVAAKVITMPDEGRP--GQMLS 107
Ttons    ---------------LALIEQYDPED-TRTSALSQPYAFVCSKVVPIGDGSEPQFNSFLS 109
Trubr    ---------------LALIEQYDPED-TRTSALSQPYAFVCSKVVPIGDGSEPQFNSFLS 109
Tverr    ---------------LALIEQYDSED-TSISALSQPYAFVCSKVVPIGDGSEPQFNFFLS 109
Abenh    ---------------LALIEQYDPED-TRISALSQPYAFVCSKVVPIGDGSEPQFNFFLS 109
Mgyps    ---------------LALIEQYDPED-TRTSTLCQPYAFVCSKVVPIGDGSEPQFNSFLS 109
Mcani    ---------------LALIEQYDPDD-TRTSTLCQPYAFVCAKVVPIGDSSAPQFTSFLS 109
Pb-01    ---------------LQLIEQYDPDD-LGADAVSQPYAYVSAKTLTMG--AKALSRAGLG 107
Pb-03    ---------------LQLIEQYDPDD-LSADAVSQPYAYVSAKTLTMG--AKALSRTGLG 107
Aderm    ---------------LQLIEQYDPED-LSPDAVSQPYAYVAAKTMTMG--AEALSGAGLG 107
Mfiji    ---------------LRFVEQYDPND-ISSSASSQPYAYVADICEEVK------------ 91
Mgram    ---------------LRFVEQYDPND-MSS--LSQPYAYVADIVEEVK------------ 89
Ptrit    ---------------LRFIEQYDPDD-LTAK--DQPYAYVCDIVQEIK------------ 89
Chete    ---------------LQFIEQYDPDD-FTVK--DQPHAYVCDIVHEIK------------ 89
Snodo    ---------------LRFIEQYDPED-LTVK--DQPYAYVCDQVHEIK------------ 89
                          . ..               .:
```

```
                                                               <----
Ncras   EERMQRLRLKLKGAEERTGERDPKANRNSGGSGGSGEYHDALEHLEESAKEKE-ETWIEQ 376
Ntetr   EERMQRLRLKLKGAEEKAGERNPKANRNSGGSGGSGEYHDALEHLEESAKEKD-ETWIEQ 377
Ndisc   EERMQ-QKQRKKGEEEKLGKRDNKANRNSGGSGGSVEFHDALEHLEEGAKEKDQMTWIEQ 406
Foxys   YDKMAG-----------------------------------------------EDGWIMK 161
Fvert   YDRMAG-----------------------------------------------EDGWIIK 161
Fgram   YERMAG-----------------------------------------------EDGWIVK 162
Trees   EALVKER---------------------------------------------NEGNWFER 170
Tvire   EALVKER---------------------------------------------NEGNWLER 165
Tatro   DALVKER---------------------------------------------NEGNWFER 174
Vdahl   EERMGRDSAGGRAMT----------------AASSDDFNNGKAGHKKGATSGKKAGWLEK 201
Moryz   QARLKESGAMGGP-------------------------HSDETGRRQRAAKDRKAGWLEK 157
Tterr   EQQQLQSARP-------------------------------AISIPSRNAPAEQPGWFEE 217
Sscle   LRFNQWS----------------------------------------------VIKLLEQ 164
Nfisc   WNPEDLAKDSP--------------------------------------LEPSAMEALTQ 127
Afumi   WNPEDLAKDSP--------------------------------------LEPSEMEALTQ 127
Aclav   WNPEDLAKDSP--------------------------------------LDPAALEALTK 127
Anige   WKPEDIFKEEP--------------------------------------LEKDAMEALTE 126
Anidu   WN-TDIFQENP--------------------------------------LDPASSEALAK 125
Aoryz   LDAEELVKESG--------------------------------------LDENAMAALTE 122
Aflav   LDAEELVKESG--------------------------------------LDENAMAALTE 122
Aterr   LDAEELVKKSG--------------------------------------LDQDAMDALTE 127
Pchry   NIEDCVEQGSG--------------------------------------LTDDQTAALEE 126
Pmarn   MEIDQKFLDDA--------------------------------------IPPEARDTLAE 126
Tstip   MNIDQRPLDDV--------------------------------------ILSDAHTALAE 126
Cimmi   LNIGEIIEKGPE-------------------------------------LSPGGPEALRR 130
Cposa   LNIGEIIEKGPE-------------------------------------LSPGGPEALRR 130
Ttons   LDLEDFISKGPG-------------------------------------LSAAELATFAN 132
Trubr   LDLEDFISKGPG-------------------------------------LSAAELATFAN 132
Tverr   LDLEDFISKGPG-------------------------------------LSAAELATFAN 132
Abenh   LDLEDFISKGPG-------------------------------------LSAAELATFTN 132
Mgyps   LDLEDFISKGPG-------------------------------------LSAEALATFAN 132
Mcani   LDLEEFISKGPG-------------------------------------LSSEALATFST 132
Pb-01   LSLQEIAQQDPG-------------------------------------LSTAGTEVFKK 130
Pb-03   LSLQEIVQLDPG-------------------------------------LSTAGTEVFKK 130
Aderm   LRVQEILQQDPG-------------------------------------LSTAGSEVFRK 130
Mfiji   LGIDIDEFRAKG-------------------------------------VGNEQWSALCE 114
Mgram   LGLDVDEVRGKE-------------------------------------VSDEQTKALAA 112
Ptrit   LGIEVDEVRGAG-------------------------------------LGEEQWAAIAE 112
Chete   LGVDIEEVQGAE-------------------------------------IADGQRAAITE 112
Snodo   TGVDFDELRGGG-------------------------------------LGEDQWAAIAD 112
                                                                 :

        ---------- E5 --------->   <-------- Acidic region ---------

Ncras   LRDELQRGEEIKWYVVVNGDEERNYSNPSDSDDEDEDEDEDE--YEYDDSEELSSSEITS 434
Ntetr   LRDELQRGEEIKWYVVVNGDEERNYSNPSDSDDEDEDEDEDEDEYEYDDSEELSSSEITS 437
Ndisc   LRDELQRGEEIKWYVVVNGDEERDYSNPSDSDDEDE-EDEDE----YDDSEDLSSSEIN- 460
Foxys   LRNELQKGEPVRWYVVVCGDDVREVPGKSDEEEKEVGYEQIRDAAQTMVRNRRTKDALEG 221
Fvert   LRNELQKGESVRWYVVVCGDDVREVPGKSDEEEKEFGYEQIRDAAQTMVRNRRTKDALEG 221
Fgram   LRDQLQKGEPVKWYVIVCGDEVREVPGKSDEEEQEVGYEQLRERAQHMARARRMREIIDG 222
Trees   LRDELQPGSPIEWYVVVNGDEERGWPHDDEEESEGS-------------------DVTEM 211
Tvire   LRDELQQGAPIEWYVVVNGDEERCVPQDDDEESFES-------------------EMREI 206
Tatro   LRDELQAGASIGWYVVVNGDEERAVPEYDDEDDEES-------------------DATE- 214
Vdahl   LRDQLQKGEDIRWYVVVCGDEERAVCEELLEAPREQ-----------------------L 238
Moryz   LRDQLQRGEEIRWYVVVNGDEERGWDHGGGDGGEGEEVDDFA-------------DGQ-- 202
Tterr   LRDQLQRGEEIRWYVVVNGDEVRDWSHEPSDRGSVSEPDSAAGYPQPPSLHRQQQDQQNY 277
Sscle   YDPDDLKTASQPWAYVSDYIVEVGLGVSISEEVKKYK----------------------- 201
Nfisc   LRDKYAAGERIGWWIVYNGDPDRAFPHSEEDDSY-DEYDYDDDDD--------------- 171
Afumi   LRDKYAAGERIGWWIVYNGDPDRAFPRYEEDDSY-DEYDYDDDDD--------------- 171
Aclav   FRDKYAAGERIGWWIVYNGDPERDYPHSEGEDSYGEDYDYDDDDDY-------------- 173
Anige   FRDKYAAGERIGWWIVYNGDPERWFPHDEEEDGDSFDDGYDEEGSYA------------- 173
Anidu   FRDKYAAGERIGWWIVYNGDPERYFPHDEDEDGMMEDDGYDD------------------ 167
Aoryz   MRDKYAAGEKIGWYIVYNGDPERWFPQIDEDDD--ESMMYDDE----------------- 163
Aflav   MRDKYAAGEKIGWYIVYNGDPERWFPQIDEDDD--ESMMYDDE----------------- 163
Aterr   FRDKYAAGEKIGWWIVYNGDPDRSFPQVEEEEE--EDESLMDE----------------- 168
Pchry   LRDRLAPGEKIGWYLVYNGDPERWYPDSEDEEYYESEEDSVDESQTRRQ----------- 175
Pmarn   IRDAIAPGAKIGWWIVYNGDPERYYPGMEDDDELIEDTEDGAGGN--------------- 171
Tstip   IRDAIAPGQKIGWWIVYNGDPERYYPGMEDD--LMED-ED-------------------- 163
Cimmi   LRDFLAPEENIGWWVVYNGDPERAFPKSDEGDSATED----------------------- 167
Cposa   LRDFLAPEENIGWWVVYNGDPERAFPKSDEGDSATED----------------------- 167
Ttons   LREVLAPGEKIGWWIVYNGNPKRFS-DKTGSDSDTTE----------------------- 168
Trubr   LREALAPGEKIRWWIVYNGNPERFSSDKTGSDSDTTE----------------------- 169
Tverr   LREVLAPGEKVGWWIVYNGNPERFSADKTGSDSDTTE----------------------- 169
Abenh   LREVLAPGEKIGWWIVYNGNPERFSSDKTGSDSDTTE----------------------- 169
Mgyps   LREVLAPGEKIGWWIVYNGNPERFSSDGDEGSDSDAE----------------------- 169
Mcani   LRDVLGPGEKIGWWIVYNGDPERFSAATEEELEHIES----------------------- 169
Pb-01   LKDELAPASEIGWFVVYNGEPERAYESSDESSSVVED----------------------- 167
Pb-03   LKDELAPASEIGWFVVYNGEPERAYENSDESSSVAED----------------------- 167
Aderm   LRNELAPDAEIGWFVVYNGDPERSFEDSDEDSPVDDG----------------------- 167
Mfiji   LRDKIAPEEKVGWYVVVCGDEDRWAPPTVG--------LLQSGGH--------------- 151
Mgram   VRDKLAPEVPVGWYVVVCGDEERLAPGNEDE---AEEEAVEAGQV--------------- 154
Ptrit   LRDKVAPGEKLGWFVVVNGDVERWAPALE----DDDTTPDASQFG--------------- 153
Chete   LRDKVAPGEKLAWFVVVNGDVERWAPPLE----DDDATPEASILS--------------- 153
Snodo   LRDKVAPGEKLGWYVVVNGDVERWAPPLESEGEDTETEATNSPFS--------------- 157
                    *  :
```

Additional Figure 2 (page 5 of 10)

```
Ncras    NYGTSYRYDTTDDLLS---TSKHPSLAA-YPPSDGRRSPLSYTGSGSRTSSRSGQSKSRS 490
Ntetr    NYGTSYRYDTTEDLLG---ASKHPSLAA-YPPSEGRRSPLSYSGSGSRTSSRGGQSKSRS 493
Ndisc    -HGTFYRHDTAEDLSASELVSKHPSLAAPYPPSEGRRSPLSYSGSASRTSSRGGQSKSPS 519
Foxys    LSDSPSEYDDDNDRDEDGEDALTVLDGILGGADGAATPRPLSRTLPDIDIPLQPQPDPNR 281
Fvert    LSDSPSEYDGDNDREEDGEDALTVLDGILGGPDGAATPRPLSRTLPDIDIPLQSQLDPSR 281
Fgram    SSNSPSEYEDDGDQDED--DALTVLEDILDHSEGASTPRPLSLELPDIHIPLQSRLDPTR 280
Trees    EDLKVAEEEDGPRREQPTGASSQMLSPTSQHAPNKPADQTHSLRH--------------K 257
Tvire    EELRVGEEESEPKREQQTAGSSRPRSTNSQNAAGRPVDQQHSLRR--------------K 252
Tatro    QGKGGDDGESEAQPEQ-------LRNKDRSQAP-RLTDQPHSLRH--------------K 252
Vdahl    EPRHIAELMRQEPVLEPAMRSFDAPRGEGHRPNKLSMDRPPEGET--------------R 284
Moryz    ---------------------------------------GD-----------SVE----D 208
Tterr    QHRYQSQYQSQAHPGRPMPTREQKQHLAQYVHQQLIFENGDREQARRPERQLALEGNDRD 337
Sscle    -------------------EKLTEEEIVPSAAEELGMSAGEIRRK--------------N 228
Nfisc    -------------------DEYTESNGGSVRNSTAPETP----------------PSP 194
Afumi    -------------------DEYTESNGDSVRNSTAPETP----------------PSP 194
Aclav    -------------------DEYTESNGESARDSTAPVTP---------------NSP 196
Anige    -------------------DGGEYESDQTQTPPETPSSA--------------------- 193
Anidu    -------------------DDDEYDRDGSSS--NTPSTP--------------------- 185
Aoryz    -------------------DGEASDYGSASQPPSTPTVS------------------ASY 186
Aflav    -------------------DGEASDYGSASQPPSTPTVS------------------ASY 186
Aterr    -------------------DEYESEDGSEQQQPSTPTVSSFD----YWCLVPMLKDHANS 205
Pchry    -------------------SQVQSQKGNRNTEPSSPQSYTVCGSFLLFSGLLALLFVLWT 216
Pmarn    -------------------DGISFHSQETGPSSVSGSMS-----------------VSVP 195
Tstip    -------------------DDINYQSRETGPSSVATSAP-----------------VKVP 187
Cimmi    -------------------DSVEQEDSERDDESKEE---------------------TGS 187
Cposa    -------------------DSVEQEDSERDDESKEE---------------------TRS 187
Ttons    -------------------GDDEPEEAGHKSGPPP----------------------ARS 187
Trubr    -------------------GDDEPEEAGHRSGPPP----------------------TRS 188
Tverr    -------------------GDGEPEEAGHRSGPPP----------------------TRS 188
Abenh    -------------------GDGEPEEAGHRSGPPVCVPIKSI-----------IIAFTHT 199
Mgyps    -------------------GGNEPEEMSHKSGQPP----------------------IRS 188
Mcani    -------------------SDDEVEEINDKSGPPL----------------------TRS 188
Pb-01    -------------------EDVVT---DSKGGIKE------------------------- 180
Pb-03    -------------------EDVVT---DRKVGIEK------------------------- 180
Aderm    -------------------EDDVMGCRDSEAATADP------------------------ 184
Mfiji    -------------------NGIRNGSEEDS------------------------------ 162
Mgram    -------------------EGQEVKGEETARESSVVTTK-----------------SFVL 178
Ptrit    -------------------NASQRSSVGQATSVQIQEPE-----------------RPST 177
Chete    -------------------PHSQASSVLQSDTSPAPEPE-----------------RPST 177
Snodo    -------------------PSSERSSVGRASDVSGQTQE-----------------RPST 181


Ncras    QSRSHSRTRTRHRNHSRPRHGKKT-RRPKPTKEQRQHHAQYTLQQELFERNDLRAS---- 545
Ntetr    QSRSHSRTRTRHRNHSRPRHGKKI-RRPKPTKEQRQHHAQYTLQQELFERNDLRAS---- 548
Ndisc    QSRSHSRTRTRHRNHSRQRHGKKISRRPKPTKEQRQHHAQYTLQQELFERNDLRASSAST 579
Foxys    ASVLTISTVWSQDESPNGTARQETPDIDAPLQPHELEKKRKPVSTASSVWSRKESLDNGL 341
Fvert    ASVLTISTVWSQDESPNGTARQETPDIDAPLQPHELDKKRKSVSTASSACSRKDSLDNAL 341
Fgram    ASVLTTSTVWSQDQSPDETANTDAPDIDKPILPSELDPNRTSMSTAYSIWSRK------- 333
Trees    ISRAGLRRLFSKKEAAE------------------------------------------- 274
Tvire    ISRAGLRRLFSKKEASE------------------------------------------- 269
Tatro    ISRAGLRRLFSKKEAPE------------------------------------------- 269
Vdahl    PRTGSRETRYSRDRSGSASKRVTGGYSPFPPPPSAAPPTHKKATSVG------------- 331
Moryz    ERRELERQELRQRLGG---IYIDE-----EDGDNYGYQVDDSSSRHTK------------ 248
Tterr    RQREQQQQQQQQQQQKPLFEKVDECRKQVQDQEQLVFETGESERERRQREYTQ------- 390
Sscle    KRAGWFDRLRQNLEMSEEMVF--------------------------------------- 249
Nfisc    AVSLALRTSLILVTDYTDGLAHSRLDSP-------------------------------- 222
Afumi    VVSLALRTSLILVTDYIDGLAHLRLDSP-------------------------------- 222
Aclav    AA-----VSLITLH---------------------------------------------- 205
Anige    --TVGLFFRLIF------------------------------------------------ 203
Anidu    --TVSLHVWLVDDALSMY------------------------------------------ 201
Aoryz    MVACSCCWHELMSVIF-------------------------------------------- 202
Aflav    MVACSCCWHELMSVIF-------------------------------------------- 202
Aterr    MNDIDVFTRKVIATLCPAKRL--------------------------------------- 226
Pchry    SSQSHCYIYMVANGVFLESPDQVLESDELGVRAVARRYETWQTSPIYSVTQCHSLPRYMC 276
Pmarn    EIPVCY--SSYYWFG--------------------------------------------- 208
Tstip    EIPVCYFLSSIQNIAQLPTLCRRILRQLLV------------------------------ 217
Cimmi    ------------------------------------------------------------
Cposa    ------------------------------------------------------------
Ttons    LKPPAVPPKPRFNLPARPKK---------------------------------------- 207
Trubr    LKPPAVPPKPRFDLPARPKK---------------------------------------- 208
Tverr    VKPPAVPPKPRFNLPARPKK---------------------------------------- 208
Abenh    VADNPVSPLDLSNLRLFPQNRGL------------------------------------- 222
Mgyps    PKPPVVPPKPKLNLPMRAKK---------------------------------------- 208
Mcani    VKPPTVPPKPRLNLPTRAKN---------------------------------------- 208
Pb-01    ------------------------------------------------------------
Pb-03    ------------------------------------------------------------
Aderm    VDSTVWSYSRSYDVKLH------------------------------------------- 201
Mfiji    AHSSTQQSHEVCGHSRCREENADHVKAN-----------------------SGDAR---- 195
Mgram    ANGASGDAERTSKASTTADEVRRHYTFN-----------------------DGRADKE-- 213
Ptrit    SRSLKKWFGG-LRKSKSGKDLRGDAAIN-TDPVPPLPPISPRHGLQSPSSSNGFAPNQGK 235
Chete    SRSLKKWFGGKLRKTKSGRSLRGDAVAAAEEPAPPLPPISPRHALP---AANAKAPEPQT 234
Snodo    SRSFKKWIGGRLKKSKSGRDLRADVPKD---PPPPLPPMSPKAAMFAPKPANGRVPLPAK 238
```

```
Ncras    -STTPTPTPTSTTTMATS-TRG-TTPTAGQAPPPPPPPTAMS-MRNTGPIIKEGAARRVV 601
Ntetr    -STAPTPTPTSTTTMATS-TRG-TTPTAGQAPPPP--PTAMS-MRNTGPIIKEGAARRVV 602
Ndisc    RSTGSTPTPTPTTTVATTNTRGGTSPTTAPAPAPAPPPTAMPAMRNTRAIIKEGAARRVV 639
Foxys    ALETPDVEMPLLHPDNTRYSASTASEIYSAYGQSRIEPEEFAKIQSSVQDPMRGEIPDVK 401
Fvert    ALETSDVEIPLLHPDNTRYSVSTASEIYSAYRQSRIEPEEFDDIHSSVQDPIRGEIPNVK 401
Fgram    --ETPDADMRLRQSEITQYSASTTSGLHSENGRTRLEPEEFAKAQPPTQNFVREETPDVV 391
Trees    ------------------------------------------------------------
Tvire    ------------------------------------------------------------
Tatro    ------------------------------------------------------------
Vdahl    ------------------------------------------------------------
Moryz    ------------------------------------------------------------
Tterr    ---------------------------------------------------------QKQ 393
Sscle    ------------------------------------------------------------
Nfisc    ------------------------------------------------------------
Afumi    ------------------------------------------------------------
Aclav    ------------------------------------------------------------
Anige    ------------------------------------------------------------
Anidu    ------------------------------------------------------------
Aoryz    ------------------------------------------------------------
Aflav    ------------------------------------------------------------
Aterr    ------------------------------------------------------------
Pchry    YTGL-------------------------------------------------------- 280
Pmarn    ------------------------------------------------------------
Tstip    ------------------------------------------------------------
Cimmi    ------------------------------------------------------------
Cposa    ------------------------------------------------------------
Ttons    ------------------------------------------------------------
Trubr    ------------------------------------------------------------
Tverr    ------------------------------------------------------------
Abenh    ------------------------------------------------------------
Mgyps    ------------------------------------------------------------
Mcani    ------------------------------------------------------------
Pb-01    ------------------------------------------------------------
Pb-03    ------------------------------------------------------------
Aderm    ------------------------------------------------------------
Mfiji    ------------------------------------------------------------
Mgram    ------------------------------------------------------------
Ptrit    NAS--------------------------------------------------------- 238
Chete    AAP--------------------------------------------------------- 237
Snodo    ISRG-------------------------------------------------------- 242


Ncras    SEGEIHPALRGQARRSEIPIIMVTPTRSTFPDETETETAGAVGEKRMVEQPQQPQPQPQQ 661
Ntetr    SEGEIHPALRGQARKSEIPIIMVTPTRSTFPDETETETAGAVGERRMVEQPQPQQPQPQS 662
Ndisc    SEEEIHPALRGVQQNPEIPIIMVTPTRSTFP--TETGMAGAAGERRMAEQQQQQQQQQQQ 697
Foxys    QPLKPNELHPSRSSTPPTPKPLQRQLPAPTIYRPESFIPPFSPVLPPMEGFDQESPRLNT 461
Fvert    QPLKPNKLHPSRSSTPPTPKPLQRQRPVPTIYRPESFVPPFSPVLPPMDGFEEVSPRPNT 461
Fgram    QPLKLAELVSSRTSTLPTPKNSQRKLPAPSLYRPESFIPPFSPPLPSSETFPDEAPKSTV 451
Trees    ------------------------------------------------------------
Tvire    ------------------------------------------------------------
Tatro    ------------------------------------------------------------
Vdahl    --------VPPAPTREPPPAREQRPATAHPQPVMESNVG--------------------- 362
Moryz    -------------------------------GPSDNGMQPLS------------------ 259
Tterr    SLPRRDLGLRERQREKEKDMANEEQPRPLFIGPGKTGMTNTTRTITTTTTTTPTKKTTTT 453
Sscle    ------------------------------------------------------------
Nfisc    ------------------------------------------------------------
Afumi    ------------------------------------------------------------
Aclav    ------------------------------------------------------------
Anige    ------------------------------------------------------------
Anidu    ------------------------------------------------------------
Aoryz    ------------------------------------------------------------
Aflav    ------------------------------------------------------------
Aterr    ------------------------------------------------------------
Pchry    ------------------------------------------------------------
Pmarn    ------------------------------------------------------------
Tstip    ------------------------------------------------------------
Cimmi    ------------------------------------------------------------
Cposa    ------------------------------------------------------------
Ttons    ------------------------------------------------------------
Trubr    ------------------------------------------------------------
Tverr    ------------------------------------------------------------
Abenh    ------------------------------------------------------------
Mgyps    ------------------------------------------------------------
Mcani    ------------------------------------------------------------
Pb-01    ------------------------------------------------------------
Pb-03    ------------------------------------------------------------
Aderm    ------------------------------------------------------------
Mfiji    ------------------------------------------------------------
Mgram    ------------------------------------------------------------
Ptrit    ------------------------------------------------------------
Chete    ------------------------------------------------------------
Snodo    ------------------------------------------------------------
```

```
Ncras    QQHQHHQQQQPQQPQPQPHPQPHQ-----------HHQINETEPGPRRGSTASANPDMGT 710
Ntetr    QQQQHHQQQQPQQPQPQPQQQPQQ-----------HHQINETEPGPRRASTASANPDMGI 711
Ndisc    QQQQQQQQQQQQQQQQQQQQQQQQQQQQQQQQQQQQQQRDEIEPETSRRGSSASN-TMGI 756
Foxys    PSRTENVKPPETSWPLSSDSPLESSGSSTFHNQDAPYPYVTSIINPKDRPGTYLNVPKEL 521
Fvert    PSRAENVKPPETSWPLSSDSPLESSGSSTFHNQDTPYPYVTSIMSPKDSLSTYLNVPKEL 521
Fgram    PNKTGATKPTEHPWPLSSDSPLGSSGSSTYHNEENSFPLVSSPAHSKNPPAATFSLPKEI 511
Trees    ------------------------------------------------------------
Tvire    ------------------------------------------------------------
Tatro    ------------------------------------------------------------
Vdahl    ------VKTPHKSRSLRRLFGMKEGSS--------------------------------- 383
Moryz    -------VMPPQDVASKPTT--------------------------KGTGS---GFRRLF 283
Tterr    PGGDRPPMVPEKDYPPRPPAGNANTLAHAGPLRPKKSVDAGAGGRPKPSGSKTGGLRRLF 513
Sscle    ------------------------------------------------------------
Nfisc    ------------------------------------------------------------
Afumi    ------------------------------------------------------------
Aclav    ------------------------------------------------------------
Anige    ------------------------------------------------------------
Anidu    ------------------------------------------------------------
Aoryz    ------------------------------------------------------------
Aflav    ------------------------------------------------------------
Aterr    ------------------------------------------------------------
Pchry    ------------------------------------------------------------
Pmarn    ------------------------------------------------------------
Tstip    ------------------------------------------------------------
Cimmi    ------------------------------------------------------------
Cposa    ------------------------------------------------------------
Ttons    ------------------------------------------------------------
Trubr    ------------------------------------------------------------
Tverr    ------------------------------------------------------------
Abenh    ------------------------------------------------------------
Mgyps    ------------------------------------------------------------
Mcani    ------------------------------------------------------------
Pb-01    ------------------------------------------------------------
Pb-03    ------------------------------------------------------------
Aderm    ------------------------------------------------------------
Mfiji    ------------------------------------------------------------
Mgram    ------------------------------------------------------------
Ptrit    ------------------------------------------------------------
Chete    ------------------------------------------------------------
Snodo    ------------------------------------------------------------


Ncras    ITSTTVPLTSSFSLDTSPSNAPTPTTIP-ALSLKPLKPPKKQSTLP----TLPTISTSSL 765
Ntetr    ITSTTVPLTSSFSLDTSPSNAPTPTTIPTGLTLKPLKPPKKNSTLPTIIPTIPTISTASL 771
Ndisc    IT-TTVPLTSSFSLDTSPSNVPTPTTIPLNLSLKTLKPLK--APKP------PKRHLSNP 807
Foxys    VPPRTPSPTSEYSPQSQNFGSNMPKFEGIVPHAPPLLVSPNPARLE--------QPSSGI 573
Fvert    VPPRTPSPTSEYSPQPQNFGSNMPKFEGIVSHAPPLLVSPNPARLE--------QPSSGI 573
Fgram    TPPRIPSPASKYPLQPQFPGPTMSRFEESVLQASPLLVTPNPKRLE--------KPSSGV 563
Trees    ------------------------------------------------------------
Tvire    ------------------------------------------------------------
Tatro    ------------------------------------------------------------
Vdahl    ------------------------------------------------------------
Moryz    GKGSKSSGDLK------------------------------------------------- 294
Tterr    GRAKADVVYAP------------------------------------------------- 524
Sscle    ------------------------------------------------------------
Nfisc    ------------------------------------------------------------
Afumi    ------------------------------------------------------------
Aclav    ------------------------------------------------------------
Anige    ------------------------------------------------------------
Anidu    ------------------------------------------------------------
Aoryz    ------------------------------------------------------------
Aflav    ------------------------------------------------------------
Aterr    ------------------------------------------------------------
Pchry    ------------------------------------------------------------
Pmarn    ------------------------------------------------------------
Tstip    ------------------------------------------------------------
Cimmi    ------------------------------------------------------------
Cposa    ------------------------------------------------------------
Ttons    ------------------------------------------------------------
Trubr    ------------------------------------------------------------
Tverr    ------------------------------------------------------------
Abenh    ------------------------------------------------------------
Mgyps    ------------------------------------------------------------
Mcani    ------------------------------------------------------------
Pb-01    ------------------------------------------------------------
Pb-03    ------------------------------------------------------------
Aderm    ------------------------------------------------------------
Mfiji    ------------------------------------------------------------
Mgram    ------------------------------------------------------------
Ptrit    ------------------------------------------------------------
Chete    ------------------------------------------------------------
Snodo    ------------------------------------------------------------
```

```
Ncras     VRNRGNRHSSTNTNTGSKRSSLTTLRSPSSWSCLSLHL---GHSH-HSTSPKDKDGEDIT 821
Ntetr     VRNRGNRHSSSNTGTGSKRSSLTTLRSPSSWSCLSLHL---GHSHSHSTSPK--DGGDTT 826
Ndisc     LSSIS--TTTTTTTTNNKRSSLTSLRSPSSWSCLSLNLNLGHHHHSTTNNPKDKDREEIN 865
Foxys     LYEAGDTISEQQLPSTPMPNAKQVALPNLDTKAA---KELVSKPSASATTPTSAHQKTIN 630
Fvert     LYEAGDTISEQRLSSTPMPKTKQVALPNLDTKAA---KEPASKP-PSATTPTSAHRKTIN 629
Fgram     VYQAGESIPKKQPPSSLPRSSVVRAPPPLNINTANSIKRSTSNPAKNITPPTSAQRETTD 623
Trees     ------------------------------------------------------------
Tvire     ------------------------------------------------------------
Tatro     ------------------------------------------------------------
Vdahl     ------------------------------------------------------------
Moryz     ------------------------------------------------------------
Tterr     ------------------------------------------------------------
Sscle     ------------------------------------------------------------
Nfisc     ------------------------------------------------------------
Afumi     ------------------------------------------------------------
Aclav     ------------------------------------------------------------
Anige     ------------------------------------------------------------
Anidu     ------------------------------------------------------------
Aoryz     ------------------------------------------------------------
Aflav     ------------------------------------------------------------
Aterr     ------------------------------------------------------------
Pchry     ------------------------------------------------------------
Pmarn     ------------------------------------------------------------
Tstip     ------------------------------------------------------------
Cimmi     ------------------------------------------------------------
Cposa     ------------------------------------------------------------
Ttons     ------------------------------------------------------------
Trubr     ------------------------------------------------------------
Tverr     ------------------------------------------------------------
Abenh     ------------------------------------------------------------
Mgyps     ------------------------------------------------------------
Mcani     ------------------------------------------------------------
Pb-01     ------------------------------------------------------------
Pb-03     ------------------------------------------------------------
Aderm     ------------------------------------------------------------
Mfiji     ------------------------------------------------------------
Mgram     ------------------------------------------------------------
Ptrit     ------------------------------------------------------------
Chete     ------------------------------------------------------------
Snodo     ------------------------------------------------------------
```

```
Ncras     GDDDDEEVVLPPPRPSPSPPIPISVN--PMG--SYSYEPVSTLPPRPAPMPPNGSNNSNK 877
Ntetr     --EMDEEVVLPPPRPSPSPPIPISLNLNPMGS-SYSYEPVSTLPPRPAPAPP-----GSK 878
Ndisc     -EEEEEEEAEDPPRPSPSPPIPLHHP-HPMGSSAYSYEPVSTLPPRPAPAPP-------K 916
Foxys     PVLKTP------PRNPATQQNTQKLPKSRLPRSVPKNIRISNVPLPPEPEIKTPRRPPTP 684
Fvert     PALRTP------PRNPTAQPTAQMSPKSRLPRSVPKNIRISNVPLPPEPEIKTPRRPPTP 683
Fgram     LNMKTPPITPITPKTPTGQPLTQASPKSRIPRSGPKTIRVSTLPPPPEPAVKSPVRPPTP 683
Trees     ------------------------------------------------------------
Tvire     ------------------------------------------------------------
Tatro     ------------------------------------------------------------
Vdahl     ------------------------------------------------------------
Moryz     ------------------------------------------------------------
Tterr     ------------------------------------------------------------
Sscle     ------------------------------------------------------------
Nfisc     ------------------------------------------------------------
Afumi     ------------------------------------------------------------
Aclav     ------------------------------------------------------------
Anige     ------------------------------------------------------------
Anidu     ------------------------------------------------------------
Aoryz     ------------------------------------------------------------
Aflav     ------------------------------------------------------------
Aterr     ------------------------------------------------------------
Pchry     ------------------------------------------------------------
Pmarn     ------------------------------------------------------------
Tstip     ------------------------------------------------------------
Cimmi     ------------------------------------------------------------
Cposa     ------------------------------------------------------------
Ttons     ------------------------------------------------------------
Trubr     ------------------------------------------------------------
Tverr     ------------------------------------------------------------
Abenh     ------------------------------------------------------------
Mgyps     ------------------------------------------------------------
Mcani     ------------------------------------------------------------
Pb-01     ------------------------------------------------------------
Pb-03     ------------------------------------------------------------
Aderm     ------------------------------------------------------------
Mfiji     ------------------------------------------------------------
Mgram     ------------------------------------------------------------
Ptrit     ------------------------------------------------------------
Chete     ------------------------------------------------------------
Snodo     ------------------------------------------------------------
```

```
Ncras    PVVSPRGGGGQTETGSNHNKVPTAPSS-PVVVKISAGPAAGPAEVQTQTQGQGQAAASPV 936
Ntetr    PVSR---GGGQVEVGSN-NKVPTAPSS-PVVVKISAGPAGPAEARRLDTKAQGQAAASPV 933
Ndisc    PASL--------------DGVPTAPSSSPVVVKISASPAEP----QTQGQGQTQAAASPV 958
Foxys    PKP--RGASGGNSQKRPQTAPEKGTPSSKAHARMLSMPYRYEAPSSLQELEREEPILEP- 741
Fvert    PKP--RGTSGGNSQKRPQTAPEKGTPSSKVHARMLSMPYRY------------------- 722
Fgram    PKPKPRLRQKEELQRRPQTAPEEGMP-LRPNGGGFSRPLQYDVPRSMQKPGGERVMLGPK 742
Trees    ------------------------------------------------------------
Tvire    ------------------------------------------------------------
Tatro    ------------------------------------------------------------
Vdahl    ------------------------------------------------------------
Moryz    ------------------------------------------------------------
Tterr    ------------------------------------------------------------
Sscle    ------------------------------------------------------------
Nfisc    ------------------------------------------------------------
Afumi    ------------------------------------------------------------
Aclav    ------------------------------------------------------------
Anige    ------------------------------------------------------------
Anidu    ------------------------------------------------------------
Aoryz    ------------------------------------------------------------
Aflav    ------------------------------------------------------------
Aterr    ------------------------------------------------------------
Pchry    ------------------------------------------------------------
Pmarn    ------------------------------------------------------------
Tstip    ------------------------------------------------------------
Cimmi    ------------------------------------------------------------
Cposa    ------------------------------------------------------------
Ttons    ------------------------------------------------------------
Trubr    ------------------------------------------------------------
Tverr    ------------------------------------------------------------
Abenh    ------------------------------------------------------------
Mgyps    ------------------------------------------------------------
Mcani    ------------------------------------------------------------
Pb-01    ------------------------------------------------------------
Pb-03    ------------------------------------------------------------
Aderm    ------------------------------------------------------------
Mfiji    ------------------------------------------------------------
Mgram    ------------------------------------------------------------
Ptrit    ------------------------------------------------------------
Chete    ------------------------------------------------------------
Snodo    ------------------------------------------------------------


Ncras    VGQYASMMSRTMPTRKGNSGNGNGNGNGNGNSGNGNGKESRRSRSSGSLRRLFQRASG 996
Ntetr    VGQYASMMSRTMPTRKGNSGN---SGNGNGNGN-----GKESRRSRSSGSLRRLFQRASG 985
Ndisc    VGQYSS-LSRTVPTRKENGSS---GGNGNGNGN-----GKESRRSRSSGSLRRLFQRASG 1009
Foxys    -----TPSLASDYNAGVSSKPNAPRDLARGVRLRAPQPVAATTQSRQTSVSTEHSDGQVN 796
Fvert    ------------------------------------------------------------
Fgram    LAPTSIPTLATSYSLSATTQANAPLNSAIPARARA-QPPSNPAQIRQPIVSNQNNDGHTL 801
Trees    ------------------------------------------------------------
Tvire    ------------------------------------------------------------
Tatro    ------------------------------------------------------------
Vdahl    ------------------------------------------------------------
Moryz    ------------------------------------------------------------
Tterr    ------------------------------------------------------------
Sscle    ------------------------------------------------------------
Nfisc    ------------------------------------------------------------
Afumi    ------------------------------------------------------------
Aclav    ------------------------------------------------------------
Anige    ------------------------------------------------------------
Anidu    ------------------------------------------------------------
Aoryz    ------------------------------------------------------------
Aflav    ------------------------------------------------------------
Aterr    ------------------------------------------------------------
Pchry    ------------------------------------------------------------
Pmarn    ------------------------------------------------------------
Tstip    ------------------------------------------------------------
Cimmi    ------------------------------------------------------------
Cposa    ------------------------------------------------------------
Ttons    ------------------------------------------------------------
Trubr    ------------------------------------------------------------
Tverr    ------------------------------------------------------------
Abenh    ------------------------------------------------------------
Mgyps    ------------------------------------------------------------
Mcani    ------------------------------------------------------------
Pb-01    ------------------------------------------------------------
Pb-03    ------------------------------------------------------------
Aderm    ------------------------------------------------------------
Mfiji    ------------------------------------------------------------
Mgram    ------------------------------------------------------------
Ptrit    ------------------------------------------------------------
Chete    ------------------------------------------------------------
Snodo    ------------------------------------------------------------
```

```
Ncras    KDGWI------------------------------- 1001
Ntetr    KEGWI------------------------------- 990
Ndisc    KEGWI------------------------------- 1014
Foxys    EDGRHPKQRIGRSHSVAEGLKKLFRKPSHAKAGHHP 832
Fvert    ------------------------------------
Fgram    EGGKHQKHHVARSHSVADGFKRLFRRPSLTKGSHH- 836
Trees    ------------------------------------
Tvire    ------------------------------------
Tatro    ------------------------------------
Vdahl    ------------------------------------
Moryz    ------------------------------------
Tterr    ------------------------------------
Sscle    ------------------------------------
Nfisc    ------------------------------------
Afumi    ------------------------------------
Aclav    ------------------------------------
Anige    ------------------------------------
Anidu    ------------------------------------
Aoryz    ------------------------------------
Aflav    ------------------------------------
Aterr    ------------------------------------
Pchry    ------------------------------------
Pmarn    ------------------------------------
Tstip    ------------------------------------
Cimmi    ------------------------------------
Cposa    ------------------------------------
Ttons    ------------------------------------
Trubr    ------------------------------------
Tverr    ------------------------------------
Abenh    ------------------------------------
Mgyps    ------------------------------------
Mcani    ------------------------------------
Pb-01    ------------------------------------
Pb-03    ------------------------------------
Aderm    ------------------------------------
Mfiji    ------------------------------------
Mgram    ------------------------------------
Ptrit    ------------------------------------
Chete    ------------------------------------
Snodo    ------------------------------------
```
